# Analysis of dermoscopy images of multi-class for early detection of skin lesions by hybrid systems based on integrating features of CNN models

**DOI:** 10.1371/journal.pone.0298305

**Published:** 2024-03-21

**Authors:** Mohammed Alshahrani, Mohammed Al-Jabbar, Ebrahim Mohammed Senan, Ibrahim Abdulrab Ahmed, Jamil Abdulhamid Mohammed Saif

**Affiliations:** 1 Computer Department, Applied College, Najran University, Najran, Saudi Arabia; 2 Department of Artificial Intelligence, Faculty of Computer Science and Information Technology, Alrazi University, Sana’a, Yemen; 3 Computer and Information Systems, Applied College, University of Bisha, Bisha, Saudi Arabia; GIET University, INDIA

## Abstract

Skin cancer is one of the most fatal skin lesions, capable of leading to fatality if not detected in its early stages. The characteristics of skin lesions are similar in many of the early stages of skin lesions. The AI in categorizing diverse types of skin lesions significantly contributes to and helps dermatologists to preserve patients’ lives. This study introduces a novel approach that capitalizes on the strengths of hybrid systems of Convolutional Neural Network (CNN) models to extract intricate features from dermoscopy images with Random Forest (Rf) and Feed Forward Neural Networks (FFNN) networks, leading to the development of hybrid systems that have superior capabilities early detection of all types of skin lesions. By integrating multiple CNN features, the proposed methods aim to improve the robustness and discriminatory capabilities of the AI system. The dermoscopy images were optimized for the ISIC2019 dataset. Then, the area of the lesions was segmented and isolated from the rest of the image by a Gradient Vector Flow (GVF) algorithm. The first strategy for dermoscopy image analysis for early diagnosis of skin lesions is by the CNN-RF and CNN-FFNN hybrid models. CNN models (DenseNet121, MobileNet, and VGG19) receive a region of interest (skin lesions) and produce highly representative feature maps for each lesion. The second strategy to analyze the area of skin lesions and diagnose their type by means of CNN-RF and CNN-FFNN hybrid models based on the features of the combined CNN models. Hybrid models based on combined CNN features have achieved promising results for diagnosing dermoscopy images of the ISIC 2019 dataset and distinguishing skin cancers from other skin lesions. The Dense-Net121-MobileNet-RF hybrid model achieved an AUC of 95.7%, an accuracy of 97.7%, a precision of 93.65%, a sensitivity of 91.93%, and a specificity of 99.49%.

## 1. Introduction

The skin plays a crucial role in maintaining overall health and well-being. The skin’s complexity and versatility make it an essential organ with various functions that contribute to the body’s homeostasis [1]. Skin lesions are abnormal growths, changes, or marks on the skin that various factors, including infections, injuries, allergies, and underlying medical conditions, cause. Prompt and accurate diagnosis of skin lesions is crucial for timely treatment and to prevent potential complications [2]. Skin lesions are triggered by many factors, including infections (bacterial, viral, or fungal), allergic reactions, exposure to sun ultraviolet, autoimmune disorders, and genetic predisposition. Treatment options depend on the type and cause of the lesion and include topical creams, surgical removal, cryotherapy, laser therapy, or systemic medications. Skin lesions are benign (non-cancerous) or malignant (cancerous) [3]. Benign skin lesions are usually harmless, but they are sometimes unsightly. Malignant skin lesions, on the other hand, are life-threatening if not treated. Sun exposure leads to various skin lesions, especially when the skin is exposed to sun ultraviolet (UV) radiation for prolonged periods without adequate protection. Some common skin lesions caused by sun exposure include Melanoma: Melanoma is the riskiest form of skin cancer and triggered by UV exposure [4]. It appears as an atypical mole with irregular borders, multiple colors, and a larger size than normal moles. Actinic Keratosis: These are rough, scaly patches that develop on sun-exposed areas of the skin, like neck, ears, face, arms, and hands. They vary in color from pink to brown and are considered precancerous, as they have the potential to develop into skin cancer if left untreated. Basal Cell Carcinoma (BCC) is the most common form of skin cancer and often develops on sun-exposed areas, such as the face, head, neck, and hands. It appears as a raised, pearly bump with visible blood vessels and bleed [5]. Various techniques are employed to diagnose skin lesions, ranging from simple visual inspection to more advanced imaging and diagnostic methods. Common techniques include: Visual Inspection: Dermatologists examine the skin with the naked eye to assess the appearance and characteristics of the lesion. Dermatoscopy involves using a handheld device with magnification and lighting to examine skin lesions in greater detail, allowing better visualization of structural features [6]. Biopsy: A sample of the lesion is taken and examined under a microscope to determine its nature. Dermatoscopy, also known as dermoscopy, is a non-invasive and valuable tool for dermatologists in the evaluation of skin lesions. It allows visualization of morphological structures not visible to the naked eye. By magnifying the lesion and reducing the surface reflection, dermatoscopy aids in identifying key features like pigmentation patterns, vascular structures, and specific dermal structures, which are crucial for accurate diagnosis [7]. Early detection of skin lesions is critical for several reasons. It enables timely intervention, prevents potential complications or spread of the lesion, and increases the likelihood of successful treatment outcomes. Regular skin checks and prompt evaluation by a dermatologist lead to the early diagnosis of skin cancers, improving the prognosis and potentially saving lives [8]. The increasing complexity of skin lesion diagnosis has led to a demand for more accurate and efficient methods. Artificial intelligence (AI) techniques has shown great promise in automating the diagnosis process. These algorithms extract deep features from dermatoscopy images and classify them accurately, helping dermatologists make better-informed decisions [9]. Identifying the type of skin lesion indeed is challenging, especially in the early stages, due to the similarity of their characteristics. Dermatologists often rely on their clinical expertise and experience to differentiate between various skin lesions. However, this time-consuming process lead to delayed diagnosis or misdiagnosis. Fortunately, advancements in technology, particularly in AI and medical imaging, offer promising solutions to aid dermatologists in improving accuracy and efficiency in skin lesion diagnosis [10]. By combining dermatologists’ expertise with AI’s power, the precision and efficiency of skin diseases diagnosis significantly enhanced. In the context of early detection, AI-powered systems aid in identifying subtle features that indicate potential malignancy or high-risk lesions [11]. This study explores the techniques for diagnosing skin lesions, focusing on dermatoscopy, the importance of early detection, and the role of artificial intelligence in improving diagnosis accuracy. The importance of combining the features of two CNN models for analyzing dermoscopy images in the early detection of skin cancer and distinguishing it from other types of skin lesions lies in its potential to enhance accuracy, robustness, and diagnostic capabilities significantly [12]. This approach capitalizes on the strengths of each individual CNN model while compensating for its limitations, thereby improving overall performance and enabling more reliable and effective diagnosis.

Some key points highlighting the significance of this feature hybrid: Different CNN architectures have varying capabilities in extracting and representing features from images. Combining features from multiple CNN models, such as DenseNet121, MobileNet, and VGG19, allows for a more comprehensive and diverse representation of the complex characteristics present in dermoscopy images. This captures subtle and prominent patterns associated with skin lesions, including skin cancer. Overfitting happens when a system becomes too specialized in the training sample and acts poorly on new, unseen data. Combining features from different CNN models mitigates the risk of overfitting. This is because each CNN model has learned different aspects of the data, and combining them helps create a more generalized and robust representation that is more effective in handling diverse cases. Skin lesions, especially in early stages, exhibit intricate and subtle variations that are challenging to distinguish using a single CNN model. Different CNN models excel in capturing certain aspects of this variability. Combining their features makes the analysis more robust and capable of handling a wider range of image variations, contributing to reliable diagnosis across diverse patient populations.

The novelty of this study in analyzing dermoscopy images from the ISIC 2019 dataset for early diagnosis of skin lesions using hybrid systems based on integrating the features of CNN models. The study introduces a novel approach by enhancing dermoscopy images using fused techniques, specifically the average filter and the CLAHE technique. The study uses a sequential feature extraction process from multiple CNN models (DenseNet121, MobileNet, VGG19). Features are merged in combinations such as DenseNet121-MobileNet, MobileNet-VGG19, and DenseNet121-VGG19. The hybridization of features from these models enables a more comprehensive and diverse representation of the complex characteristics present in skin lesions. The combination of features from multiple CNN models facilitates the capture of subtle and prominent patterns associated with skin lesions, including those indicative of skin cancer. This approach enhances the system’s ability to represent dermoscopy images’ intricate details and variations. The merging of features from CNN models leads to the formation of high-dimensional and potentially duplicate features. To address this, the study introduces the application of the t-SNE algorithm. This dimensionality reduction technique is employed to select the most important and representative features for each class (lesion) in the ISIC 2019 dataset, promoting efficiency in subsequent analysis.

The primary contributions of this study encompass the following aspects:

- Applying nested techniques to improve dermoscopy images by averaging filter and CLAHE method.
- Representing the features of high-dimensional CNN models in low-dimensional areas by the t-SNE algorithm, thus reducing the features of CNN.
- Applying a hybrid method between CNN models and RF and FFNN algorithms for early diagnosis of skin lesions for the ISIC2019 data set.
- Applying a hybrid method of RF-CNN and FFNN-CNN algorithms based on the fusion of features of CNN models for the early diagnosis of skin lesions of the ISIC2019 data set.

The rest of the paper is organized as follows: Section 2 delves into the methodologies and outcomes derived from previous studies. Section 3 presents the approaches employed in analysing dermoscopy images to facilitate timely identification of skin lesions. Section 4 provides an exposition of the results yielded by the hybrid models. The performance evaluation and comparative analysis of the systems are detailed in Section 5. Finally, Section 6 encapsulates the paper with concluding remarks.

## 2. Related work

The section reviews previous studies, materials and methods, and results for analyzing dermoscopy images to detect skin lesions.

Hatice et al. [13] presented a new deep learning-based CNN called InSiNet, designed for the detection of benign and malignant skin lesions. The efficacy of this method was evaluated using ISIC 2019 dataset. They compared InSiNet’s performance against several other machine learning techniques. The InSiNet model achieved an accuracy of 91.89%. Seyed et al. [14] an automated system for melanoma detection was developed using an ensemble approach that combined CNNs and image texture feature extraction. In the feature extraction-based phase, texture features were extracted to enhance classification performance. The study evaluated this method on ISIC 2019, in which the method’s performance metrics were 96.7% accuracy, 95.1% average precision, 96.3% sensitivity, and 97.1% specificity. Hadi et al. [15] a preprocessing method was presented for the extraction of the region of interest (RoI) from skin lesions. The experimental findings indicated that training CNN models with the RoI-extracted dataset led to enhanced prediction accuracy. Employing the InceptionResNetV2 architecture showed a notable improvement of 2.18% in accuracy. These outcomes collectively demonstrate the efficacy of the proposed RoI extraction technique in improving classification accuracy while also expediting the computational processes associated with training and evaluating CNN classifiers. Junsheng et al. [16] proposed a few-shot segmentation method for segmenting skin lesions, minimizing the need for extensive pixel-level annotations. The methodology involves several sequential steps. The co-occurrence region was derived from both the support and query images. Subsequently, the outcomes of this process are combined and forwarded to an inference module for the purpose of predicting the query image’s segmentation. The results affirm the method’s potential as a promising framework for achieving accurate few-shot segmentation of skin lesions, offering a practical solution to the resource-intensive data annotation challenges inherent in this domain. Syed et al. [17] investigated CNN were harnessed, employing the surrogate gradient descent technique to classify a dataset of ISIC 2019. The model yielded an accuracy of 89.57%, surpassing the performance of both VGG-13 and AlexNet architectures while utilizing fewer trainable parameters. Fayadh et al. [18] presented a WT-DRNNet model for classifying skin lesion, employing a combination of wavelet transform-based preprocessing and a deep residual neural network architecture. Deep features were extracted using a residual neural network through transfer learning. The model achieved accuracy, precision, and F1-Score metrics of 95.73%, 95.84%, and 93.44% for the HAM10000 dataset, respectively. Imran et al. [19] a DCNN model was meticulously crafted with multiple layers and diverse filter sizes, yet employs fewer filters and parameters to enhance effectiveness and performance. The DCNN approach were achieves precision of 94%, specificity of 91%, sensitivity of 93%in the ISIC dataset. Selen et al. [20] presented an approach combining Swin Transformer and CNNs is presented for the task of multiclass skin lesion classification. The issue of class imbalance is mitigated through the utilization of a weighted cross-entropy loss. Notably, the proposed approach achieves impressive performance metrics, including sensitivity (82.3%), specificity (97.9%), accuracy (97.2%), and balanced accuracy (82.3%). Zillur et al. [21] presented an approach for classifying types of skin lesions through a weighted average model. Class balancing, nois and removal methods to enhance model performance. The results of the evaluation reveal strong performance from the individual models, with macro-average recall scores of 91%, and 84% achieved by DenseNet, Xception, and ResNet. In the study of Mostafiz et al [22] a digital technique for hair removal from skin images is introduced, involving morphological filtering and an inpainting algorithm. The Grabcut technique is used to isolate lesions. Extracting features from skin images involves the utilization of the GLCM and statistical features techniques. Irfan et al. [23] introduced an automated model for diagnosing and prioritizing skin cancer cases, investigating the impact of incorporating clinical features. They employ an ensemble-learning approach, combining the EfficientNetB3 model for skin lesion analysis with Extreme Gradient Boosting for clinical data integration. The model achieved the performance of an accuracy of 78%, precision of 89%, recall of 86%, and F1 score of 88%. Qilin et al. [24] presented Grad-CAM to enhance accuracy during the inference stage for classifying skin lesions based on dermoscopic images, aiming to categorize them into various diagnostic classes provided by the ISIC 2018 dataset. The findings indicate that incorporating additional metadata into the model significantly enhances classification performance. The algorithm reached a balanced multiclass accuracy of 88.7%. Natasha et al. [25] introduced an explainable artificial intelligence (XAI) system for classifying skin lesions, aiming to enhance classification accuracy and support dermatologists in early skin cancer diagnosis. The system reached an accuracy of 94.47% and recall of 94.01%. Arias et al. [26] presented a framework to assess the classification efficacy of CNNs in discerning skin diseases. The CNNs used transfer learning, leveraging pre-trained weights from the ImageNet dataset. The most proficient model was evaluated on the ISIC2019 dataset, resulting in an accuracy of 93%.

The previous study used CNN models and machine learning to classify skin images from the ISIC 2019 dataset. However, they did not achieve satisfactory accuracy. Previous studies have not applied hybrid methods that integrate features from different sources. Previous research has not effectively addressed incorporating hybrid methodologies that amalgamate features, which are pivotal for effectively tackling the resemblance of clinical indications and achieving encouraging outcomes. This is important because it helps to address the similarity of clinical signs and improves classification accuracy. This study was distinguished by its comprehensive hybrid methodologies and tools, situated at the hybrid machine learning and deep learning paradigms. The core of this approach revolved around integrating attributes from multi-CNN models features, then classified by RF and FFNN networks.

## 3. Materials and methods

### 3.1. ISIC 2019 dataset description

The proposed methodologies underwent training and evaluation using the publicly accessible ISIC 2019 dataset, which caters to researchers and individuals interested in medical diagnosis and predictive analysis. The ISIC 2019 dataset comprises 25,331 images sourced from the amalgamation of the HAM10000 and BCN_20000 datasets [27]. This dataset is notable for furnishing high-quality images with accompanying metadata encompassing lesion location, gender, and age of the affected individuals. Within the ISIC 2019 dataset, there are 10,015 RGB images with a resolution of 600 x 450 pixels, while the BCN_20000 subset encompasses RGB images at 1024 x 1024 pixels. The division of the dataset is delineated across eight classes representing various types of skin lesions: 628 images belonging to Squamous Cell Carcinoma (SCC), 867 images depicting Actinic Keratoses (AKIEC), 3323 images representing Basal Cell Carcinoma (BCC), 2624 images characterizing Benign Keratosis Lesions (BKL), 239 images showcasing Dermatofibroma (DF), 4522 images portraying Melanoma (MEL), 12,875 images encompassing Nevi (VN), and 253 images capturing Vascular lesions (VASC). The application of this dataset extended to diverse hybrid technologies grounded in hybrid attributes. The key goal of this study is to facilitate early identification of the types of skin lesions, particularly discerning melanoma from other types [28].

### 3.2. Enhancement images of ISIC 2019 dataset

The enhancement of ISIC 2019 dermoscopy images is important for several reasons, primarily related to dermatology and skin cancer diagnosis. Enhancing dermoscopy images help reveal finer details and structures within skin lesions, making it easier for AI techniques to accurately diagnose various skin conditions, including melanoma and other types of skin cancer. Enhancing images improves the quality of image analysis and reduces the chances of misdiagnosis due to poor image quality or artifacts. Enhanced images help identify subtle changes in lesion characteristics that might not be apparent in standard images, enabling earlier intervention [29]. Enhancing ISIC 2019, dermoscopic images involve various image processing techniques, such as: Removing or reducing unwanted noise or artifacts from the images to improve the clarity of features. Adjusting the contrast to make subtle features and textures more distinguishable aids in identifying lesions. Enhancing the sharpness of the image to make fine details more visible is crucial for accurate diagnosis. Eliminating any artifacts introduced during the imaging process as hair could obscure important diagnostic features.

In this study, to improve the ISIC 2019 Dermoscopic Images, an averaging filter was applied to remove noise and the Contrast limited adaptive histogram equalization (CLAHE) technique was applied to increase the contrast of the edges of the skin lesions. An average filter is used to reduce noise in an image through smoothing out pixel values. It replaces the value of each pixel in the image with the average value of its neighboring pixels. The size of the filter, often referred to as the "kernel size," determines how many neighboring pixels are considered in the averaging process. In this work, a 5x5 average filter was used for each pixel in the image, a 5x5 neighborhood (25 pixels in total) around it is considered [30]. The average value of these 25 pixels is then computed, and the central pixel’s value is replaced with this average value. This process is repeated for every pixel in the image, effectively reducing noise as Eq 1. The step-by-step explanation of how the average filter works with a 5x5 kernel: Place the center of the 5x5 kernel over the first pixel of the image. Take the pixel values within the kernel (a 5x5 square area centered on the current pixel). Calculate the average of these pixel values. Assign the calculated average value to the current pixel’s position. Move the kernel to the next pixel and repeat the process until you’ve covered the entire image. The result is a smoother version of the original image with reduced high-frequency noise.


$$F(y)=\frac{1}{N}\sum_{k=0}^{N-1}z(y-k)$$ (1)

F(y) represents the resulting outcome, N denotes the number of pixels within operators, z(y) pertains to the input, and z(y-k) pertains to the preceding input.

The choice of the kernel size for an average filter, such as 5x5, is a common practice in image processing and enhancement tasks, including the enhancement of dermoscopy images from the ISIC 2019 dataset. The kernel size determines the spatial extent to which the filter operates on the image. The reasons to select a 5x5 kernel size. A larger kernel size results in a stronger smoothing effect on the image. In the context of dermoscopy images, smoothing helps reduce noise and fine-scale variations, making the image visually more coherent. While smoothing is desirable, it’s crucial to avoid excessive blurring that could lead to loss of important details and edges in dermoscopy images. A 5x5 kernel balances noise reduction and preservation of essential structures. Larger kernel sizes generally require more computational resources. A 5x5 kernel is a reasonable compromise between achieving the desired smoothing effect and maintaining computational efficiency. A 5x5 kernel allows for both local and global enhancement. Local details are preserved, and global smoothing helps enhance the image’s overall appearance, which is beneficial in dermoscopy images where certain features need to be highlighted. Dermoscopy images may contain artifacts or small irregularities that are effectively mitigated by using a 5x5 average filter. This size is often sufficient to eliminate small-scale noise while retaining important image features.

**Fig 1:**
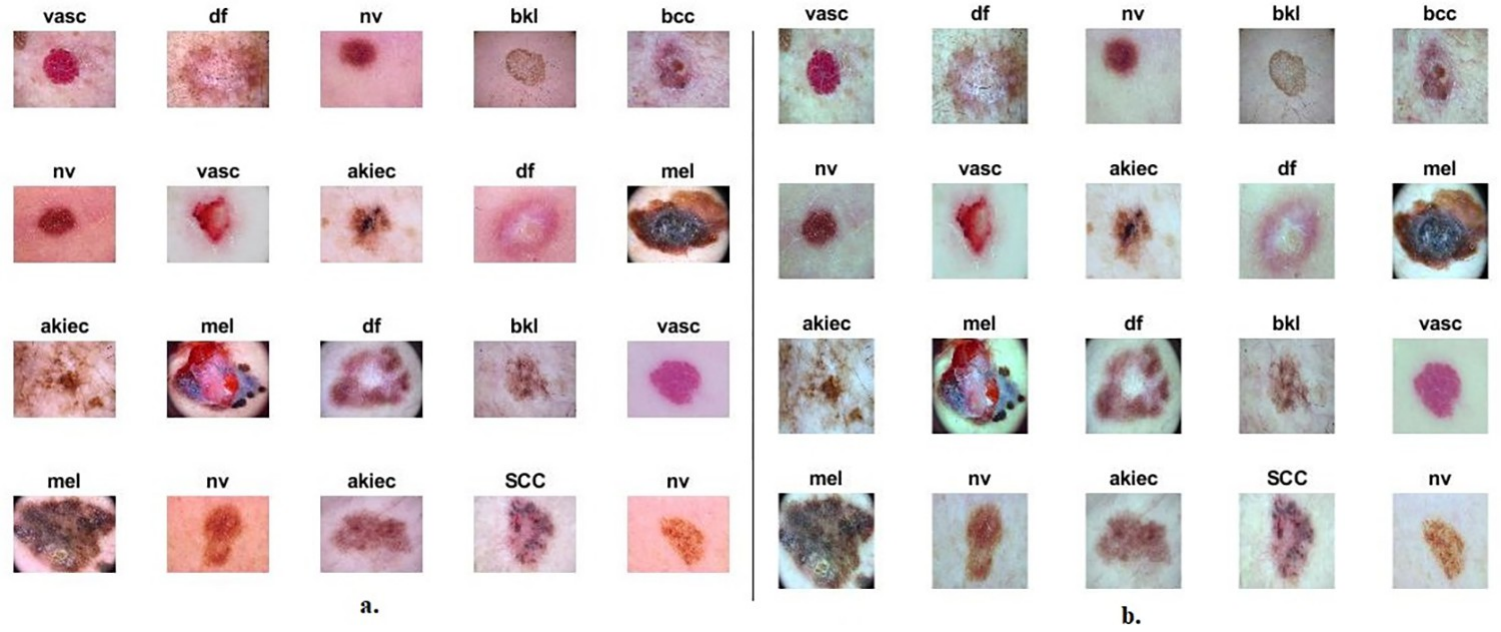
Images of the ISIC 2019 dataset a. original images b. after enhancement.

Because of the subdued differentiation between the boundaries of skin lesions and the surrounding areas, the CLAHE method was employed to ameliorate this diminished contrast. The CLAHE algorithm is used to enhance the contrast of dermoscopy images of the ISIC 2019 dataset, particularly to reveal low-contrast lesion areas. CLAHE is an extension of the traditional histogram equalization method. Histogram equalization enhances the contrast of an image by redistributing pixel intensities in the histogram to cover the full intensity range. However, in the case of dermoscopy images, which may have varying illumination conditions, applying global histogram equalization might lead to overamplification of noise [31]. CLAHE addresses adaptive blocks by dividing the image into smaller, non-overlapping blocks or tiles. Histogram equalization is then applied independently to each block. This adaptive approach allows for better preservation of local contrast, ensuring that the enhancement is tailored to the characteristics of specific regions within the image. To prevent overamplification of noise in regions with low contrast, CLAHE introduces a contrast-limiting mechanism. After histogram equalization is applied to each block, a contrast enhancement factor is computed. If the factor exceeds a predefined limit, pixel intensities in the block are scaled down. This limiting step ensures that extreme amplification is constrained, maintaining a balance between contrast improvement and noise control. After processing all blocks independently, the enhanced blocks are reassembled to reconstruct the final image. The result is an image with improved contrast, where the details in low-contrast lesion areas are more perceptible. In the context of dermoscopy images from the ISIC 2019 dataset, where low-contrast lesion areas may be challenging to discern, CLAHE acts as an effective tool to reveal subtle details. It adapts to the local characteristics of the image, enhancing contrast where needed without introducing excessive noise. This iterative process extends to each pixel within the image until the enhancement of skin lesion edges becomes evident. As illustrated in Fig 1B, representative instances from the ISIC 2019 dataset display the outcomes after applying these optimization techniques. Illustration 1. b, displays examples extracted from the ISIC 2019 dataset after improvement methods.

### 3.3. Gradient vector flow algorithm

Separating the skin lesions from the surrounding regions within the images is critical in extracting features from the region of the lesions only. This process, often referred to as image segmentation, plays a critical role in the identification and analysis of specific regions of interest, particularly in the context of skin lesions. In this investigation, the application of the GVF technique aimed to delineate the lesion region from the surrounding image context, facilitating subsequent analytical processes within the realm of image processing [32]. The GVF method is a significant technique for segmenting skin lesions from ISIC 2019 Dermoscopic Images. It enables the isolation and separation of these lesions from the surrounding parts of the image. The GVF method capitalizes on the directional information provided by the gradient vectors of the image, facilitating precise localization of boundaries between different regions. The gradient vector at each pixel in an image is calculated to capture the rate of change of pixel intensity. This is often computed using partial derivatives in the x and y directions as Eq 2.

$$f(x,y)=-|\nabla [G\sigma (x,y)*I(x,y)]{|}^{2}$$ (2)

where the symbol ∇ denotes the gradient operator and *Gσ*(*x*,*y*) represents a two-dimensional Gaussian function with a standard deviation of σ.

The GVF field is obtained by evolving the gradient vectors over multiple iterations [33]. This field assists in capturing the prominent edges and boundaries within the image. The flow operates to reduce the energy functionalization within the gradient vector via the GVF approach. The energy functional is contingent upon image smoothing and the extent of edge noise inherent in the image. The magnitude of image edge noise is quantified by parameter μ. When confronted with elevated edge noise, augmenting parameter μ becomes advisable, resulting in diminished noise along the edges and a concomitant attenuation of contour strength. The GVF field is represented as a vector function as Eq 3:

$$GVF\;field\;(F)=\mu *\nabla^{2}V+\nabla V$$ (3)

Where ∇^2^ represents the Laplacian operator, and μ is a parameter controlling the influence of the Laplacian term.

**Fig 2:**
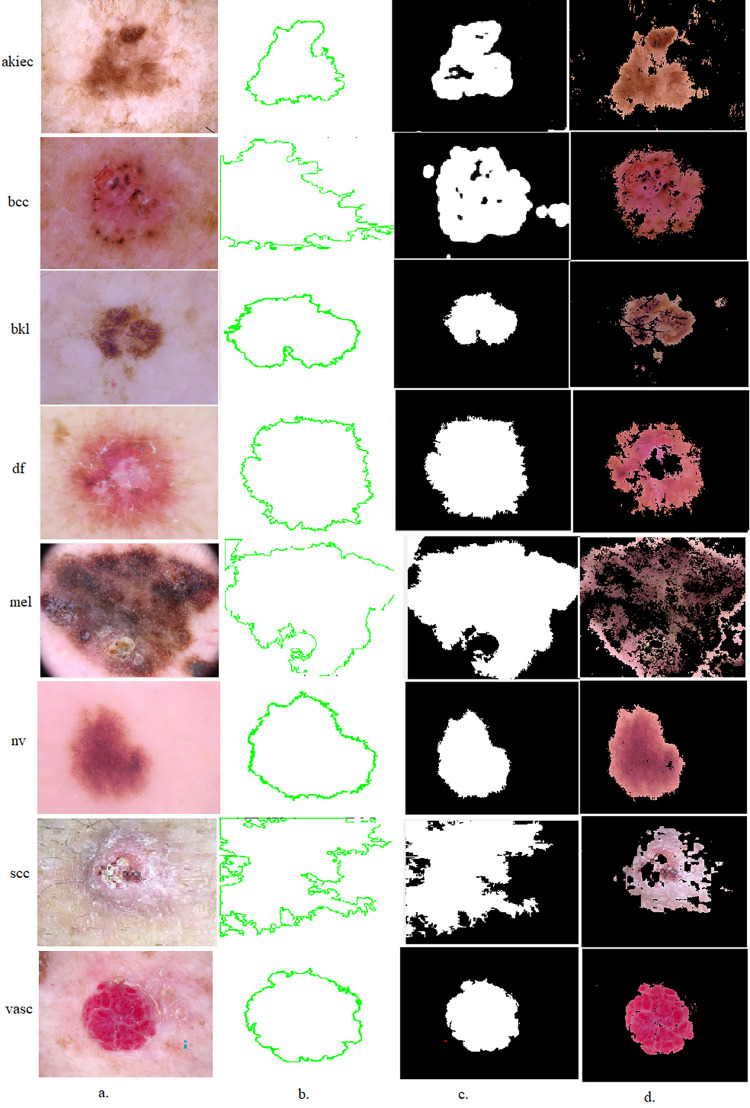
Dermoscopy images of the ISIC 2019 dataset for lesion isolation from healthy skin by GVF algorithm a. original images b. Selected lesion region c. Lesion region segmentation d. Lesion interest (ROI).

The GVF field is then employed to evolve the initial contour or boundary of the skin lesion. This is typically done using a deformable contour model like the Snake model. The contour is attracted towards the strong edges and boundaries the GVF field identifies. The GVF method’s effectiveness lies in its ability to capture intricate details and handle complex boundaries present in skin lesions. By utilizing the gradient vector information and evolving the contour according to the GVF field, the method provides more accurate segmentation results than traditional edge-based methods. The delineation of the impacted region is achieved using parameters through gradient vector flow models. These models define the boundaries of the affected regions and separate them from the remainder of the image. The objective of employing this GVF algorithm is to separate regions of infection and segregate them from the adjacent healthy areas called Region of Interest (ROI), facilitating subsequent comprehensive analysis. This approach constitutes a significant contribution to the study, as it involves segmenting and storing all affected areas from the ISIC2019 dataset dermoscopy images in a novel folder named ISIC2019-ROI. Then inputted ISIC2019-ROI dataset into CNN models containing dermoscopy images of the skin lesion. Consequently, the CNN models are focused exclusively on analyzing dermoscopy images depicting areas of infection, circumventing the need to process complete images encompassing healthy regions. Fig 2 visually illustrates a subset of the dataset that underwent segmentation, highlighting the specific selection of affected areas.

### 3.4. Extract deep feature

CNNs comprise numerous convolutional and pooling layers, culminating in fully connected layers. The convolutional layers process an input image with dimensions m * n * z, where ’m’ and ’n’ denote the image’s width and height, and ’z’ represents the number of color channels [34]. The quantity of convolutional layers varies across different networks, with each convolutional layer typically comprising multiple filters sized f * f * z. It’s essential for the input image’s channel count to match that of the convolutional filter. At the CNNs’ core is the convolution process, wherein the filter f(t) is overlaid onto the input image x(t). This filter is systematically shifted across the image until all image areas are covered; this step is crucial as the filter must analyze every image pixel. The output of the convolution operation is a feature map, which represents the image that highlights the presence of specific features. This process is mathematically represented as Eq 4. Each individual convolutional layer generates feature maps, with the count of feature maps equating to the number of filters present within that layer. The resultant image is relayed to the subsequent layer after undergoing the convolutional layers’ operations. This progression involves introducing biases and routing the image through auxiliary layers like Rectified Linear Units (ReLU) for further processing. The number of convolutional layers in a CNN vary [35]. The initial layers capture basic features at a lower level, such as edges and corners. Subsequent layers then discern more complex features at a higher level, such as objects and shapes. In a CNN, the last layer usually takes the form of a fully connected layer. This standard neural network layer establishes connections between all neurons in the preceding layer and all neurons within the final layer. The role of the fully connected layer involves the categorization of the input image into a class [36].


y(t)=(x*f)(t)=∫x(a)f(t−a)da (4)

Where f (t) represents the filter, x(t) represents the input image, and y(t) represents the resulting output.

Pooling layers in CNNs serve as a mechanism to reduce the feature generated by convolutional layers while retaining the most salient information. This process aids in controlling computational complexity, mitigating overfitting, and enabling the network to focus on essential features, in the context of analyzing dermoscopy images from the ISIC 2019 dataset. Feature maps are generated after passing the dermoscopy images through one or more convolutional layers [37]. These feature maps highlight different visual patterns and structures the convolutional filters detect. Pooling involves dividing each feature map into non-overlapping or partially overlapping regions [38]. These pooling regions are typically small, such as 2x2 or 3x3. Within each pooling region, a pooling operation is performed to aggregate information. The most common pooling operations are max-pooling and average-pooling: Max-Pooling: The maximum value within the pooling region is selected in this operation. Max-pooling effectively captures the most dominant features within the region, as shown in Eq 5 [39]. Average-Pooling: Here, the average value of the pooling region is computed. Average pooling helps maintain more general information about the region, as shown in Eq 6. The pooling operation results in a reduced dimensionality feature map. Since pooling regions are smaller than the original input, the output of the pooling operation is a down sampled representation of the input features.

$$z(i;\;j)={max}_{m,n=1\ldots .k}f[(i-1)p+m;\;(j-1)p+n]$$ (5)

$$z(i;\;j)=\frac{1}{k^{2}}\sum_{m,n=1\ldots .k}f[(i-1)p+m;\;(j-1)p+n]$$ (6)

where f denotes the filter applied to the area image, while m, n indicate the specific position within the matrix, the parameter k represents the number of pixels, and p signifies the step size used.

In the final stage of the process, the primary goal is to classify the input data. This entails providing probabilities for each potential class to which the input could belong. To achieve this, the CNN architecture employs fully connected layers, transforming the complex and abstract features extracted from the earlier layers into more compact and manageable vectors. These vectors represent the distinctive characteristics of the input and serve as the basis for making accurate class predictions. Once the high-level features have been translated into vectors, the SoftMax activation function is employed [40]. This function operates through a set of neurons, each corresponding to a specific class. The number of neurons in this layer corresponds to the number of classes in the classification problem. The SoftMax function performs a mathematical operation that converts the computed scores into normalized probabilities. Essentially, SoftMax assists in associating the extracted features of an image with the most appropriate class label.

In the last layers of the DenseNet121, MobileNet, and VGG19 models, specifically within the final convolutional or pooling layers, the computational process results in more advanced feature maps. These feature maps possess dimensions that reflect the relationships and patterns extracted from the input images. For DenseNet121, the dimensions are (16, 32, 512), for MobileNet, they are (7, 7, 512), and for VGG19, they are (7, 7, 1024). These dimensions essentially represent these feature maps’ spatial organization (height, width) and depth (number of channels). Following this, a global average pooling layer comes into use. This layer transforms the feature maps from the previous stages into feature vectors [41]. This is achieved by computing the average value within each channel of the feature map, effectively flattening the spatial dimensions. For DenseNet121 and MobileNet, this process results in feature vectors of size 1024, while for VGG19, the feature vectors have a size of 4096. These feature vectors encapsulate the key high-level information extracted from the input images, providing a concise, well-suited representation for subsequent processing. Considering the entire ISIC 2019 dataset, which contains 25,331 images, these feature vectors are organized into a matrix where each row corresponds to an image, and the columns contain the extracted features. For DenseNet121 and MobileNet models, the resulting matrix has dimensions 25,331 x 1024, indicating that a vector of length 1024 represents each image. In the case of the VGG19 model, the matrix is of size 25,331 x 4096, signifying a longer feature vector of 4096 dimensions for each image. This features matrix serves as the input to RF and FFNN algorithms, enabling the algorithms to detect lesions on the ISIC 2019 dataset.

The selection of DenseNet121, MobileNet, and VGG19 models for the analysis of dermoscopy images in the ISIC 2019 dataset for early diagnosis of skin lesions is caused by several reasons: DenseNet121, MobileNet, and VGG19 represent diverse architectures in terms of depth, connectivity, and computational efficiency. Utilizing models with distinct architectures allows for a comprehensive exploration of feature extraction capabilities. DenseNet121 is known for its dense connectivity patterns, which promote feature reuse and are beneficial for capturing intricate patterns in dermoscopy images. MobileNet is designed for efficiency, particularly on mobile devices, making it suitable for resource-constrained applications. VGG19, with its deep and traditional architecture, excels at learning hierarchical features. DenseNet121, MobileNet, and VGG19 operate at different levels of abstraction. DenseNet121 with dense connectivity captures fine-grained details, MobileNet focuses on efficient feature extraction, and VGG19 with deeper layers learn more abstract representations. This enables a comprehensive analysis of dermoscopy images at various scales. DenseNet121, MobileNet, and VGG19 are well-established models with documented performance on dermoscopy datasets. Analyzing dermoscopy images is a challenging task that benefits from a comprehensive evaluation. By using multiple models with diverse architectures, the ISIC 2019 dataset gains insights into the types of features each model captures and how they contribute to the diagnostic process.

### 3.5. T-Distributed stochastic neighbor embedding algorithm

The t-SNE algorithm works to reduce the dimensions of features of high-dimensional DenseNet121, MobileNet, and VGG19 models by selecting the most important skin lesions-related features and deleting redundant and unimportant features: The first step is to calculate the pairwise similarities between all data points in the high-dimensional space. This is done by using a Gaussian kernel to smooth the distances between the points. The probability of two points being neighbors is then calculated as in Eq 7 [42].

$$p(x_{i},x_{j})=\exp\left(-\frac{∥x_{i}-x_{j}{∥}^{2}}{\left(2*{sigma}^{2}\right)}\right)$$ (7)

where *x*_*i*_, *x*_*j*_ are the two points, ||*x*_*i*_−*x*_*j*_|| is the distance between the two points, and sigma is a parameter that controls the bandwidth of the Gaussian kernel.

The second step is using pairwise similarities to create a probability distribution over all data points. This is done by normalizing the pairwise similarities. The t-SNE algorithm then iteratively maps the high-dimensional features to a lower-dimensional space in a way that preserves the probabilities of the points being neighbors. This is done by minimizing the following cost as in Eq 8.

$$KL=\sum_{i}\sum_{j}P(x_{i},x_{j})log\frac{P({x_{i}/x}_{j})}{Q(x_{i},x_{j})}$$ (8)

where P(x_i_, x_j_) is the probability of two points being neighbors in the high-dimensional space, Q(x_i_, x_j_) is the probability of two points being neighbors in the lower-dimensional space, and KL is the Kullback-Leibler divergence.

The third step is to map the data points to a low-dimensional space using a t-SNE transformation to maintain the local form of the data. The fourth step is selecting the features with the highest values in the low-dimensional space [43]. These features are most important for representing the data in the low-dimensional space. The t-SNE algorithm is able to select the most important features by iteratively moving the points in the lower-dimensional space so that the points that are most similar in the high-dimensional space are also most similar in the lower-dimensional space. This process is repeated until the algorithm converges. The fifth step is to delete the features with the lowest values in the low-dimensional space [44]. These features are not important for representing the data in low-dimensional space. The redundant and unimportant features are then eliminated by selecting the features that have the highest values of Q(x_i_, x_j_).

The original extensive features extracted from DenseNet121, MobileNet, and VGG19 models underwent a dimensionality reduction, resulting in 724, 694, and 921 feature dimensions, respectively. Therefore, the data set became represented by a feature matrix of size 25331 x 724, 25331 x 694 and 25331 x 921 for DenseNet121, MobileNet, and VGG19 models, respectively. In this context, the original complex and high-dimensional features derived from the mentioned neural network architectures were effectively compressed or reduced in size while retaining essential information. As a result, the dataset was reformatted into a more manageable representation characterized by matrices containing a specific number of rows (25331, reflecting the dataset size) and a reduced number of columns (724, 694, or 921, corresponding to models). This process allows for more efficient analysis and interpretation of the data, as it simplifies the computational load and enhances the ability to uncover patterns and relationships within the dataset.

### 3.6. Inductive and deductive phase

The final phase of medical image processing is classification, a process intricately linked to the efficacy of preceding stages. Following the stages involving refinement and delineation of the ROI, specifically the lesion area, the inherent attributes of skin lesions are extracted utilizing CNN models. These attributes are then structured into vectors for storage. The aggregate attributes of the dataset find representation in a feature matrix, which serves as the input for both RF and FFNN networks. The classification networks undertake an inductive phase to construct a classification model, termed "data training." This involves the network learning patterns and relationships within the provided data to create a model capable of categorizing skin lesions effectively. Subsequently, the deductive stage involves assessing the system’s performance by subjecting it to new data and measuring its ability to classify skin lesions accurately. This process is crucial in evaluating the system’s applicability and effectiveness [45].

#### 3.6.1. Random forest algorithm

The Random Forest algorithm is an ensemble learning technique that enhances the precision and resilience of classification or regression assignments by amalgamating numerous decision trees. The fundamental concept underlying the Random Forest approach involves the construction of a collection of decision trees, each trained on a random subset of the data and its attributes. The resulting predictions of these trees are then aggregated their predictions to make a final prediction. Randomly sample (with replacement) a subset of the original training data. This is known as the "bootstrap" sample. Every individual decision tree within the forest undergoes training using a bootstrap sample. Each decision tree randomly selects a subset of features from the total feature set [46]. This is done to introduce diversity among the trees and reduce the risk of overfitting. Using the sampled data and features, a decision tree is constructed. The algorithm selects the best feature and split point at each tree node. The steps are repeated until multiple decision trees are generated in the forest with different smoothing models and feature subsets. Each decision tree in the forest makes a prediction for classification tasks, and the final class label is determined by a majority vote among the trees (the most common predicted class). Each decision tree predicts a numerical value for regression tasks, and the final prediction is the average of the predicted values from all trees. The key strengths of the RF algorithm include: By aggregating the predictions of multiple decision trees, the algorithm reduces the risk of overfitting, a common issue in single decision trees [47]. The randomness introduced through data and feature sampling helps the model generalize to new, unseen data. RF is less sensitive to outliers and noise in the data than single decision trees. The construction of individual decision trees is parallelized, making RF suitable for large datasets. This work applies the RF methodology to input datasets of dimensions 25331 x 724, 25331 x 694, and 25331 x 921, derived from three models: DenseNet121, MobileNet, and VGG19.

#### 3.6.2. Feed forward neural networks

FFNN consist of multiple tiers of interconnected nodes (neurons), structured into an input layer for inputs, followed by one or more hidden layers and an output layer. The input layer accommodates neurons that align with the data’s input features. Each neuron represents a single feature, and the number of neurons in this layer matches the dimensionality of the input data [48]. In this work, fifteen hidden layers, each hidden layer contains multiple neurons that compute intermediate representations of the input data. Neurons in the hidden layers apply a linear transformation (weighted sum of inputs) followed by an activation function. The activation function presents characteristics to the model, enabling it to acquire intricate associations within the data. The output layer produces the final predictions for the network. The number of neurons in the output layer depends on the number of classes of the dataset. Each class contains a neuron in a multiclass classification. In this work, the number of neurons is eight neurons. During forward propagation, input data is fed into the network through the input layer. Every neuron in the successive layers calculates a weighted summation of its input values with a bias factor and then subjects the outcome to the activation function. This sequence of steps iterates through each layer until the final layer is reached, generating the ultimate predictions [49]. A loss function quantifies the difference between the predicted outputs and target values through mean squared error (MSE). The objective of the network is to reduce the value of the loss function by modifying the weights and biases associated with the neurons. The FFNN is trained by repeatedly cycling through the training data, performing forward propagation to compute predictions and backward propagation to update the model’s parameters. This process continues until the model converges, achieving a satisfactory level of performance. Once the FFNN is trained, it is used to make predictions on new, unseen data by performing forward propagation through the trained network. In this work, the FFNN algorithm is applied to input datasets of dimensions 25331 x 724, 25331 x 694, and 25331 x 921, generated from three models: DenseNet121, MobileNet, and VGG19. The FFNN algorithm proceeds to perform an important step known as dataset partitioning. This testing phase assesses the FFNN’s ability to generalize its predictions to new, unseen data. The division of the dataset into these subsets allows the FFNN to be trained and evaluated on separate data, which is critical for assessing its performance and generalization capabilities. By allocating a portion of the data exclusively for testing, the study ensures that the FFNN’s performance is evaluated to simulate its real-world application on new. This approach helps mitigate the risk of overfitting, where the FFNN perform well on the training data but struggle to generalize to new data.

### 3.7. Proposed systems training

#### 3.7.1. Training of pre-trained strategies

The first methodology of this research involved employing pre-trained DenseNet121, MobileNet, and VGG19 architectures to extract and categorize features from skin lesions of the ISIC2019 dataset. This methodology encompassed the utilization of image enhancement methods, notably applying an Average filter for noise reduction and implementing the CLAHE technique to enhance appear of contours within low-contrast lesions. After applying these image processing techniques, the dataset underwent optimization before being fed into the pre-trained models. The convolutional strata of these models undertook the extraction of salient attributes present within the skin lesions. This process entails the systematic movement of filters across images, facilitating the identification of pertinent features and the generation of feature maps. The pooling layers were employed to reduce the dimensions of these feature maps. The output from the pooling layers was subsequently directed to fully connected layers, operating in a classifier capacity. These layers scrutinized the identified attributes and subsequently executed the classification of the input images.

#### 3.7.2. Training of hybrid systems

**Fig 3:**
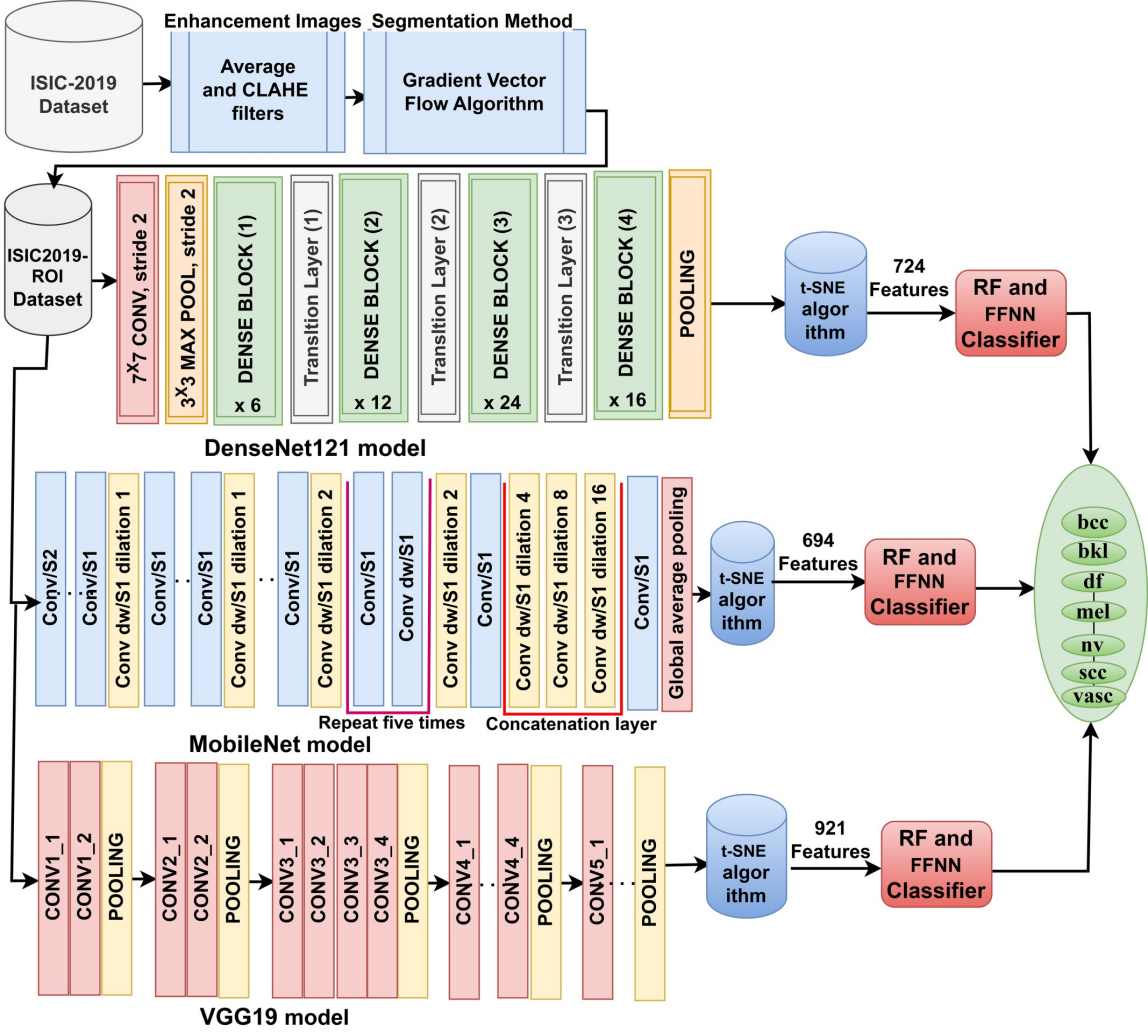
Analysis of ISIC 2019 dataset image for skin lesions detection using a hybrid technique.

This work presented a novel hybrid methodology designed to analyse dermoscopy images with the specific goal of early diagnosis of skin lesions. The procedural framework is elucidated in Fig 2, encompassing the following operational stages:

In first phase, the dermoscopy images undergo enhancement via the Average filter and CLAHE technique. This process serves a dual purpose: noise reduction and image contrast enhancement, both crucial for accurate subsequent analysis. The second step involves the application of the GVF algorithm. This algorithm isolates the lesion areas of interest from the image by delineating the boundaries of the lesions for accurate subsequent analysis. The lesion areas from the ISIC 2019 dataset are analysed through established neural network architectures: DenseNet121, MobileNet, and VGG19. These models analyse the lesion areas, extracting intricate features that are crucial information for diagnostic purposes. The resultant features are compiled into feature matrices 25331 x 724, 25331 x 694, and 25331 x 921 for DenseNet121, MobileNet, and VGG19. The ensuing stage entails the employment of the t-SNE algorithm. This algorithm facilitates the transformation of the high-dimensional feature matrices into a lower-dimensional representation, which is pivotal for analysis, enabling the identification of patterns and relationships that might be obscured in higher dimensions. And extracting the most important representative features strongly correlates with skin lesions’ features. The last phase involves feeding the low-dimensional feature matrices—dimensionally into RF and FFNN classifiers. This study presents an innovative approach amalgamating advanced image enhancement techniques, state-of-the-art neural network architectures, dimensionality reduction, and machine learning classifiers. This comprehensive methodology demonstrates its potential for early skin lesion detection and is a promising avenue for advancing dermatological diagnostic practices as shown in Fig 3.

#### 3.7.3. Training of hybrid systems based on fusion CNN features

**Fig 4:**
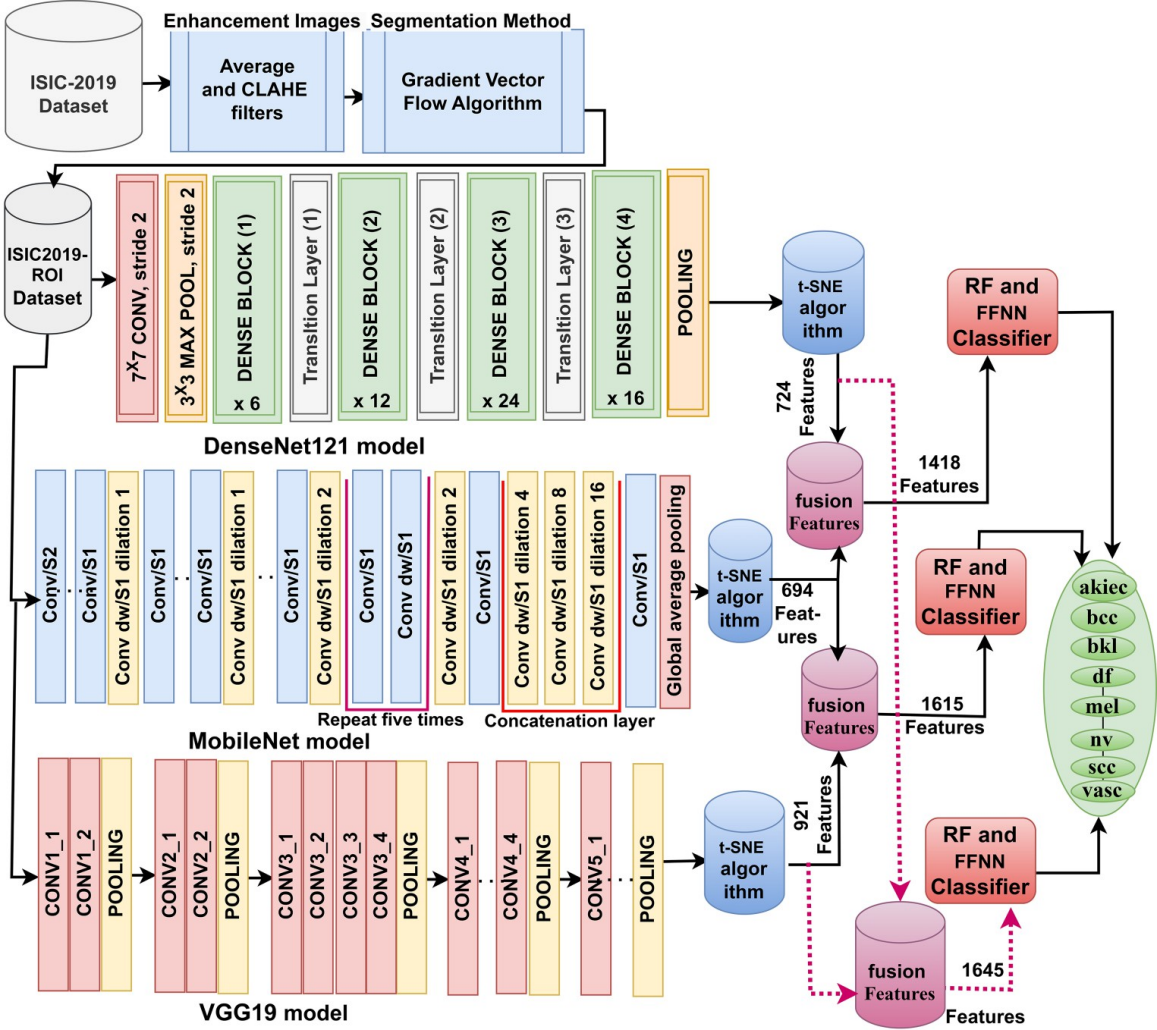
Analysis of ISIC 2019 dataset image for detecting skin lesions using a hybrid technique based on fused features of CNN models.

This paper presented a novel approach for the early identification of skin lesions using dermoscopy images. The approach combines elements from CNN models, specifically DenseNet121, MobileNet, and VGG19, with the classification capabilities of RF and FFNN architectures. The step-by-step process is illustrated in Fig 4.

The first four steps in this strategy are the same as the previous strategy. The fifth step involves fusing CNN models’ features: DenseNet121-MobileNet, MobileNet-VGG19, and DenseNet121-VGG19. These features are then kept in feature vectors. In the sixth step, the reduced-dimensional hybrid feature matrix, with dimensions of 25331 x 1418 for DenseNet121, 25331 x 1615 for MobileNet, and 25331 x 1645 for VGG19, is inputted into both the RF and FFNN networks. This step is crucial as it facilitates the classification of skin lesions based on the combined features obtained from the hybrid CNN models.

## 4. Experimental results

### 4.1. Splitting of ISIC 2019 dataset

The performance assessment of the novel methodologies outlined in this research was conducted using dermoscopy images sourced from the ISIC 2019 dataset, which is readily accessible to researchers and domain experts. The ISIC 2019 dataset encompasses a total of 25,331 dermoscopy images, categorized into eight classes corresponding to various types of skin diseases as detailed in Table 1. The data set was divided into 60% for training and validation (90:10) and the remaining 40% was kept for the testing phase. Allocating 40% to the testing phase for several reasons: First, data augmentation technology will increase the training data set, which leads to a large training data set. Second, the proposed systems have a high ability to generalize to other data sets.

**Table 1:** Splitting the ISIC 2019 data sets of skin lesion.

Classes	Training	validation	Testing
Scc	339	38	251
Akiec	468	52	347
Bcc	1795	199	1329
Bkl	1417	157	1050
Df	129	14	96
Mel	2442	271	1809
Nv	6952	773	5150
Vasc	137	15	101

Notably, the dataset’s distribution among classes is uneven, leading to an imbalanced dataset, as some classes contain more images than others, which affect the accuracy of the methods as accuracy tends to have majority classes.

### 4.2. Performance measures

The confusion matrix is an essential assessment tool for evaluating the efficacy of classification systems applied to a given dataset. A confusion matrix is a table that helps us understand how well a classification model is performing. It summarizes a dataset’s predicted and actual classes, enabling us to calculate various performance metrics. The confusion matrix is formed with rows and columns of equal length, corresponding to the different classes within the dataset. Within this matrix are entries denoting the count of accurately and inaccurately classified instances belonging to a particular test group. Notably, the principal diagonal of the matrix embodies accurately classified instances, termed as true positives (TP), while the remaining cells encompass instances that have been inaccurately classified, comprising true negatives (TN) and false negatives (FN). The effectiveness of these systems is quantified by utilizing Eqs 9 to 13. These equations take their variables from the values within the confusion matrix.

The Area Under the Curve (AUC) is a common metric used to evaluate the performance of multi-class classification systems, proposed for analyzing dermoscopy images in the context of early diagnosis of skin lesions of the ISIC 2019 dataset. The Receiver Operating Characteristic (ROC) curve is the graphical representation of the relationship between the true positive rate (sensitivity) and the false positive rate (1-specificity) across different thresholds. The ROC curve plots the true positive rate (sensitivity) against the false positive rate (1-specificity) for different classification thresholds. Each point on the curve represents a trade-off between sensitivity and specificity.The AUC is a scalar value representing the area under the ROC curve. A higher AUC indicates better discriminative ability, with a value of 1.0 suggesting perfect classification and 0.5 indicating random chance. AUC provides a comprehensive assessment of diagnostic accuracy without being affected by the choice of a specific threshold. It considers the model’s ability to discriminate between classes across all possible decision thresholds. A high AUC suggests that the proposed system effectively distinguishes between skin lesions, which is crucial for early diagnosis. The ROC curve allows for visualising the sensitivity (true positive rate) and specificity (true negative rate) across different decision thresholds. A well-performing system has high sensitivity to correctly identify skin lesions (true positives) and high specificity to minimize false positives.


$$AUC=\frac{TP\;Rate}{FP\;Rate}$$ (9)

$$Accuracy=\frac{TN+TP}{TN+TP+FN+FP}*100\%$$ (10)

$$Precision=\frac{TP}{TP+FP}*100\%$$ (11)

$$Sensitivity=\frac{TP}{TP+FN}*100\%$$ (12)

$$Specificity=\frac{TN}{TN+FP}*100$$ (13)

### 4.3. Balancing and augmenting the ISIC 2019 dataset

**Fig 5:**
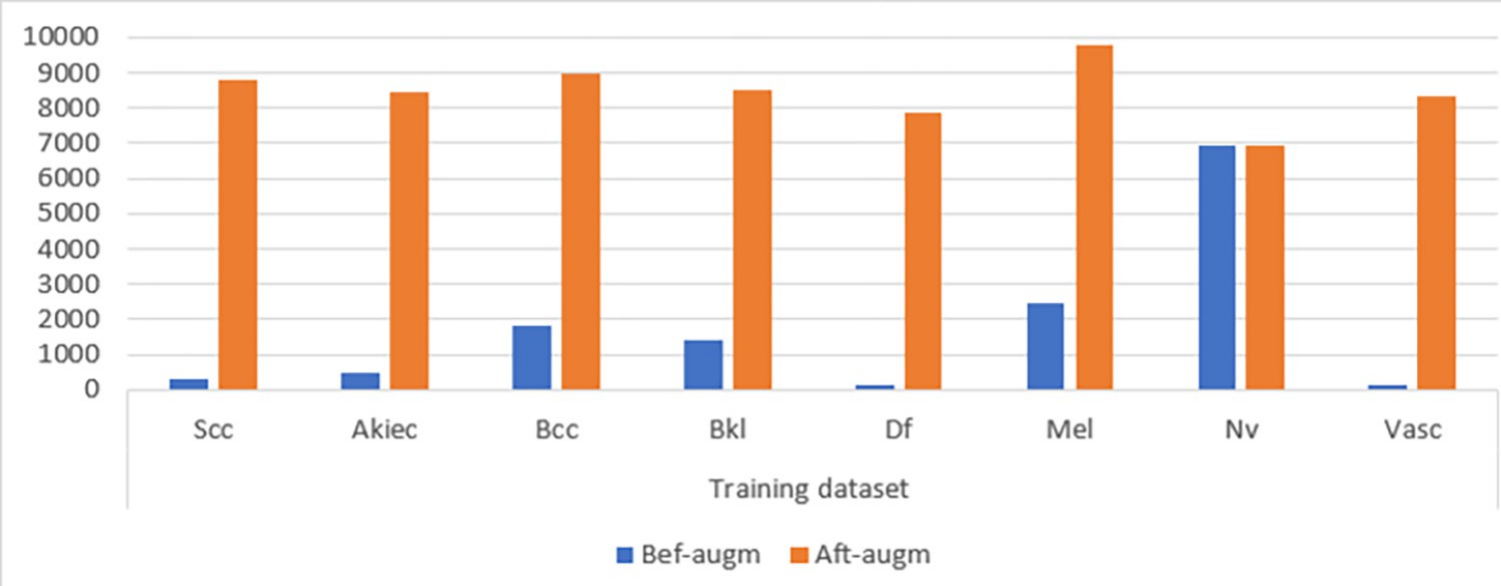
Data augmentation performance presentation of the ISIC 2019 dataset before and after the implementation of the technology.

CNN encounter several difficulties, such as overfitting and imbalanced datasets. Overfitting occurs when a model learns the training data too well and cannot generalize to new data. This happens when the model is trained on a small data set or when the data set is not diverse enough. Unbalanced data sets contain classes with different numbers of samples. This led to the model learning only to classify the majority class well, while the minority classes are not classified as well. To address these challenges, CNN models use a data augmentation tool. Data augmentation is a method that artificially enlarges the training dataset’s magnitude by generating novel instances derived from the existing samples. This is done by applying random transformations to the images, such as rotating, resizing, flipping, and cropping. Data augmentation aims to create a more diverse and challenging training data set that will help the model generalize better to new data. In biomedical data sets, data augmentation is particularly important because these data sets are often small and unbalanced. The ISIC 2019 training dataset for skin cancer classification contains 16,211 images, unevenly distributed into eight classes. The meticulous system employed to rectify the class imbalance is noteworthy: augmentation is applied to images across seven classes, orchestrated to harmonize the class distribution. Intriguingly, the "nevi" class remains untouched due to its satisfactory volume of images. Each class undergoes augmentation by a factor, a calculated endeavor to achieve equilibrium. The empirical results, illustrated in Table 2 and Fig 5, conspicuously present the transformation wrought by the data augmentation tool on the ISIC 2019 training dataset. A discernible pattern emerges, revealing the extent of augmentation for each class: the "Scc" class experiences a twenty-five-times increase, the "Akiec" class expands by 17 times, the "Bcc" class expands by class four times, the "Bkl" class expands by class five times, "Df" class undergoes a sixty-time augmentation, "Mel" class expands by three times, and "Vasc" class expands by sixty-time augmentation. The data augmentation method used in the study increased the number of training dataset images. The number of images in each class has been increased by a different number depending on the number of original images in a method that achieves a balance with other classes. The study results showed that data augmentation improved the performance of the CNN model on the ISIC 2019 test data set.

**Table 2:** Balancing and augmenting the ISIC 2019 dataset for skin lesions.

Phase	Training dataset							
Classes	Scc	Akiec	Bcc	Bkl	Df	Mel	Nv	Vasc
Bef-augm	339	468	1795	1417	129	2442	6952	137
Aft-augm	**8814**	**8,424**	**8,975**	**8,502**	**7869**	**9,768**	**6952**	**8357**

### 4.4. Results of pre-trained CNNs

This section overviews the outcome of the pre-trained DenseNet121, MobileNet, and VGG19 models. These models underwent training using the ImageNet dataset, a collection comprising over 1.2 million images designed for classifying more than 1,000 categories. Regrettably, the ImageNet dataset lacks the inclusion of a majority of biomedical image datasets, including dermoscopy images of skin lesions. Nonetheless, these models leverage the knowledge acquired during their training on the ImageNet dataset to tackle novel tasks involving the classification of dermoscopy images. The first layers of these models receive input in the form of skin lesion images sourced from the ISIC 2019 dataset. These images then undergo processing through convolutional, pooling, and auxiliary layers, extracting intricate and latent features. Subsequently, fully connected layers transform these higher-level features into vectors and classify each resultant feature vector into the suitable classification class.

**Fig 6:**
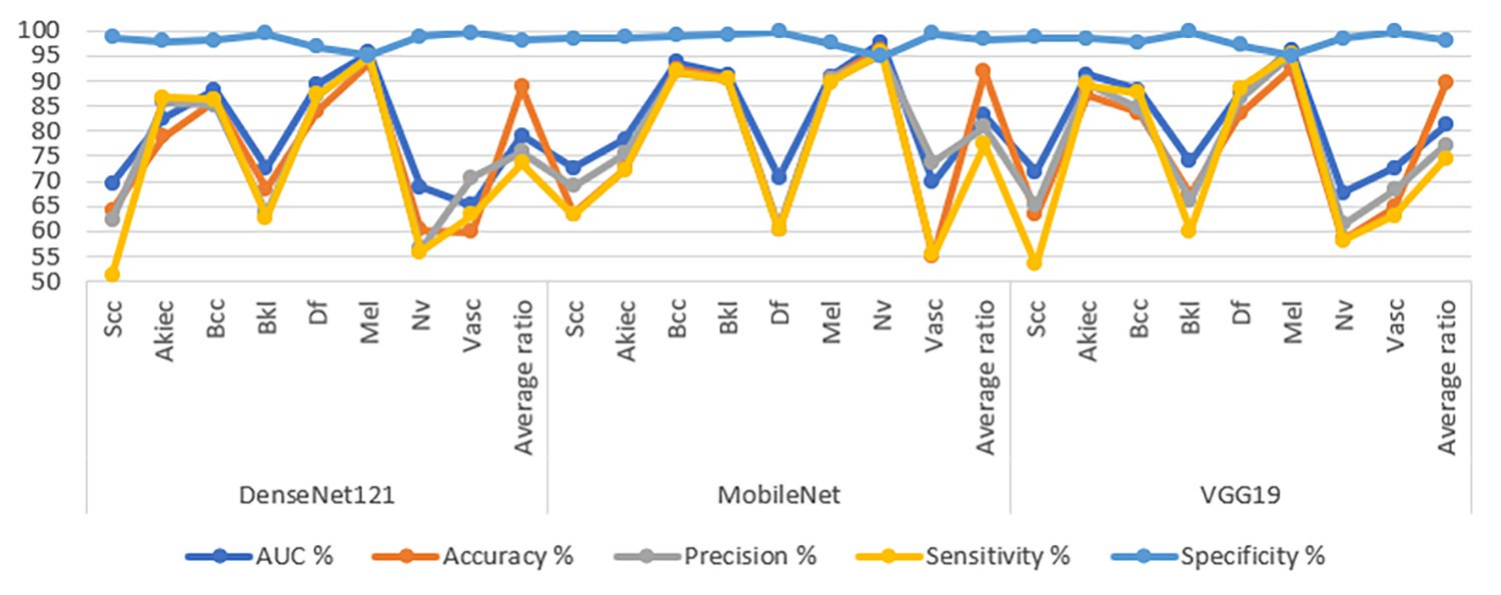
Display performance of CNN models for image analysis of the ISIC2019 dataset for early detection of skin lesions.

Table 3 and Fig 6 summarize the performance of three pre-trained models, DenseNet121, MobileNet, and VGG19, on the task of diagnosing skin lesions in dermoscopy images from the ISIC 2019 dataset. The DenseNet121 has achieved an AUC of 79.01%, accuracy of 88.79%, precision of 75.78%, sensitivity of 73.54%, and specificity of 98.06%. The MobileNet has achieved an AUC of 83.15%, accuracy of 91.8%, precision of 80.83%, sensitivity of 77.31%, and specificity of 98.41%. The VGG19 has achieved an AUC of 81.15%, accuracy of 89.6%, precision of 77.14%, sensitivity of 74.46%, and specificity of 98.18%.

**Table 3:** Results of pre-trained CNN models for early diagnosis skin lesion.

Models	Classes	AUC %	Accuracy %	Precision %	Sensitivity %	Specificity %
DenseNet121	Scc	69.5	64.2	62.3	51.2	98.7
	Akiec	82.6	78.9	85.8	86.7	97.9
	Bcc	88.2	86.2	85.2	86.4	98.2
	Bkl	72.7	68.5	63.7	62.8	99.5
	Df	89.3	84.1	87.1	87.2	96.7
	Mel	95.7	93.7	94.9	94.9	95.1
	Nv	68.9	60.3	56.4	55.8	98.8
	Vasc	65.2	59.8	70.8	63.3	99.6
	**Average ratio**	**79.01**	**88.79**	**75.78**	**73.54**	**98.06**
MobileNet	Scc	72.6	63.5	69	63.2	98.5
	Akiec	78.4	72.4	75.4	72.3	98.7
	Bcc	93.8	92.5	91.9	91.8	99.1
	Bkl	91.2	90.5	90.3	90.4	99.3
	Df	70.8	60.4	60.4	60.2	99.8
	Mel	90.9	90.5	90.6	89.7	97.6
	Nv	97.8	96.3	95.3	95.6	94.8
	Vasc	69.7	54.9	73.7	55.3	99.5
	**Average ratio**	**83.15**	**91.8**	**80.83**	**77.31**	**98.41**
VGG19	Scc	71.9	63.2	65.2	53.4	98.7
	Akiec	91.2	87.3	89.6	89.2	98.5
	Bcc	88.3	83.6	84.7	87.7	97.7
	Bkl	73.9	67.2	66.2	60.1	99.8
	Df	87.4	83.5	86.6	88.6	97.2
	Mel	96.3	92.7	95.1	95.4	95.1
	Nv	67.6	58.3	61.4	58.2	98.6
	Vasc	72.6	64.9	68.3	63.1	99.8
	**Average ratio**	**81.15**	**89.6**	**77.14**	**74.46**	**98.18**

### 4.5. Results of hybrid systems of deep learning, RF, and FFNN

The section presents hybrid models that emerge from the fusion of CNN architectures, namely DenseNet121, MobileNet, and VGG19, with both RF and FFNN networks. This fusion is conducted independently to scrutinize skin lesion images within the confines of the ISIC 2019 dataset. The underlying mechanism of these hybrid systems revolves around a twofold process: first, the segmentation of the lesion region is performed; subsequently, feature maps are extracted through the utilization of the CNN models. Importantly, the t-SNE technique is harnessed to sift through the amassed feature vectors. This involves retaining salient attributes while discarding superfluous ones. The ensuing stage involves channelling the feature vectors, which have been refined via the t-SNE approach, into the RF and FFNN networks. These networks are then subjected to training and subsequent performance assessments. Remarkably, the amalgamation of CNN with RF and FFNN networks, herein referred to as CNN-RF and CNN-FFNN hybrid models, is demonstrated to exhibit remarkable proficiency in the image analysis of skin lesions. Notably, these hybrid models possess an inherent ability to discern skin malignancies from other types of skin ailments, underscoring their robust discriminatory capabilities in the realm of clinical diagnosis and disease differentiation.

**Fig 7:**
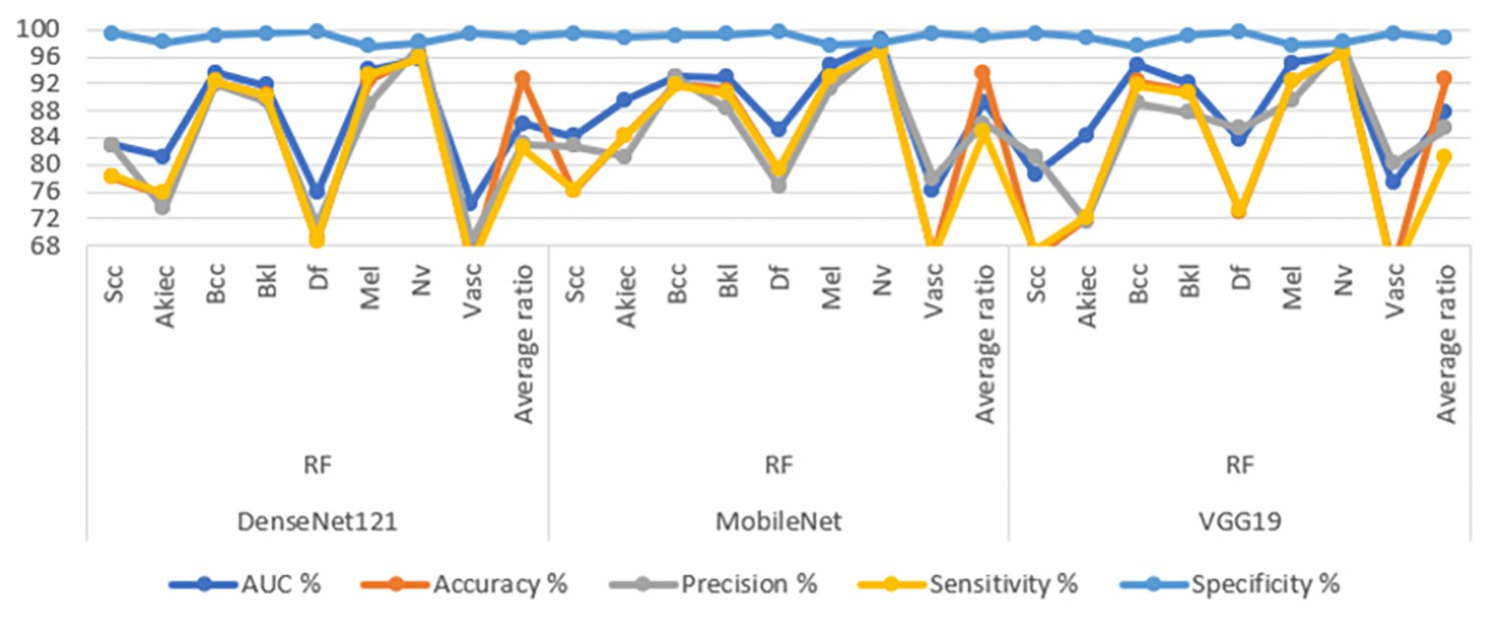
Display performance of hybrid system of CNN-RF for image analysis of the ISIC2019 dataset for early detection of skin lesions.

Table 4 and Fig 7 comprehensively present outcomes using CNN-RF hybrid models to analyse dermoscopy images for the early detection of skin lesions. The ensuing details elaborate on the results obtained for each model within this hybrid framework. The CNN-RF hybrid model, upon evaluation, has demonstrated exceptional performance. The DenseNet121-RF model achieved an AUC of 86.15%, accuracy of 92.8%, precision of 83.01%, sensitivity of 82.44%, and specificity of 98.89%. The MobileNet-RF model achieved an AUC of 89.25%, accuracy of 93.6%, precision of 86.08%, sensitivity of 84.81%, and specificity of 99%. The VGG19-RF model yielded an AUC of 87.76%, accuracy of 92.6%, precision of 85.31%, sensitivity of 80.94%, and specificity of 98.79%.

**Table 4:** Results of the hybrid system of CNN-RF for diagnosis of skin cancer.

Combined features	Classifier	Classes	AUC %	Accuracy %	Precision %	Sensitivity %	Specificity %
DenseNet121	RF	Scc	82.9	78.1	82.7	78.3	99.5
		Akiec	81.2	75.5	73.4	75.8	98.2
		Bcc	93.6	92.2	91.9	92.3	99.1
		Bkl	91.8	90.3	89.5	90.1	99.4
		Df	75.8	68.8	71	68.6	99.7
		Mel	94.1	92.5	89	93.4	97.5
		Nv	95.6	96.4	97.8	95.8	98.2
		Vasc	74.2	65.3	68.8	65.2	99.5
		**Average ratio**	**86.15**	**92.8**	**83.01**	**82.44**	**98.89**
MobileNet	RF	Scc	84.2	76.1	82.7	76.2	99.5
		Akiec	89.6	84.4	81.2	84.3	98.9
		Bcc	93.1	91.9	92.9	91.8	99.2
		Bkl	92.9	91	88.3	90.6	99.3
		Df	85.1	79.2	76.8	79.3	99.7
		Mel	94.6	92.8	91.2	93.1	97.7
		Nv	98.4	97.1	97.6	96.8	98.2
		Vasc	76.1	66.3	77.9	66.4	99.5
		**Average ratio**	**89.25**	**93.6**	**86.08**	**84.81**	**99**
VGG19	RF	Scc	78.6	66.5	81.1	67.2	99.5
		Akiec	84.3	72	71.6	72.1	98.8
		Bcc	94.8	92.4	89.1	91.8	97.6
		Bkl	92.1	90.7	87.7	90.6	99.2
		Df	83.6	72.9	85.4	73.2	99.8
		Mel	95.1	92.4	89.5	92.4	97.7
		Nv	96.4	96.6	97.9	96.5	98.2
		Vasc	77.2	64.4	80.2	63.7	99.5
		**Average ratio**	**87.76**	**92.6**	**85.31**	**80.94**	**98.79**

The hybrid compositions involving both CNN-RF and CNN-FFNN generate confusion matrices, which display the effectiveness of these hybrid models in the early detection of skin cancers, specifically in distinguishing skin cancers from other types of lesions.

**Fig 8:**
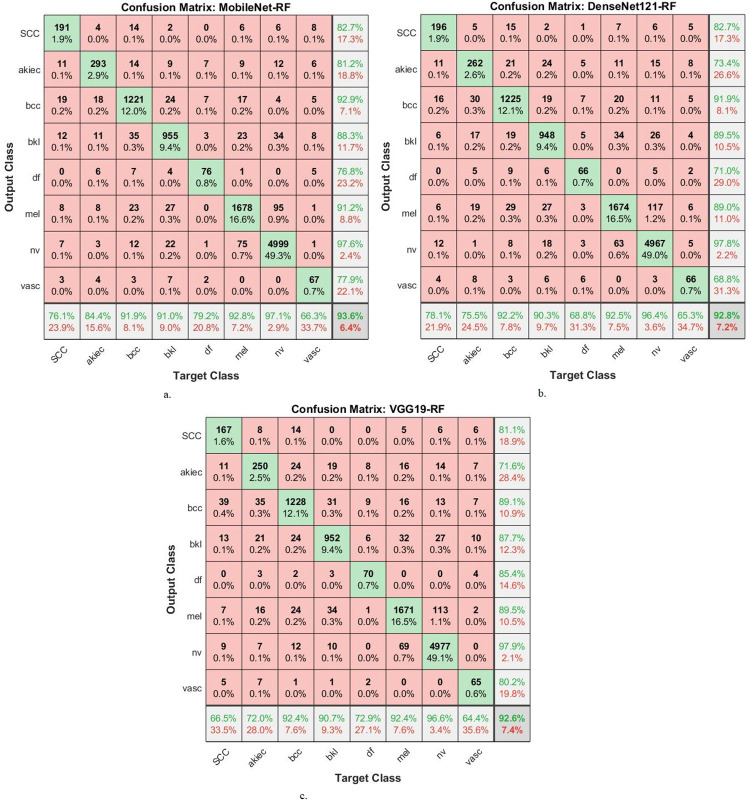
Confusion matrix for Display performance of hybrid system of CNN-RF for dermoscopy image analysis of the ISIC2019 dataset for early detection of skin lesions a. DenseNet121-RF b. MobileNet-RF c. VGG19-RF.

Fig 8 provides a graphical representation of the confusion matrices generated by the MobileNet-RF, DenseNet121-RF, and VGG19-RF models for early diagnosis of skin lesions. The figure furnishes insight into the accuracy attained by each class within the classification framework. Specifically, the MobileNet -RF model achieves a remarkable accuracy across all classes, with the following results: 76.1% accuracy for the Scc class, 84.4% for Akice class, 91.9% for Bcc class, 91% for Bkl class, 79.2% for Df class, 92.8% for Mel class, 97.1% for Nv class, and 66.3% for Vasc class. Similarly, the DenseNet121-RF model displays an accuracy across all classes. It achieves 78.1% accuracy for the Scc class, 75.5% for Akice class, 92.2% for Bcc class, 90.3% for Bkl class, 68.8% for Df class, 92.5% for Mel class, 96.4% for Nv class, and 65.3% for Vasc class. Furthermore, the VGG19-FFNN model portrays perfect accuracy rates for each class. This is evident from the results, where it obtains 66.5% accuracy for the Scc class, 72% for Akice class, 92.4% for Bcc class, 90.7% for Bkl class, 72.9% for Df class, 92.4% for Mel class, 96.6% for Nv class, and 64.4% for Vasc class.

**Fig 9:**
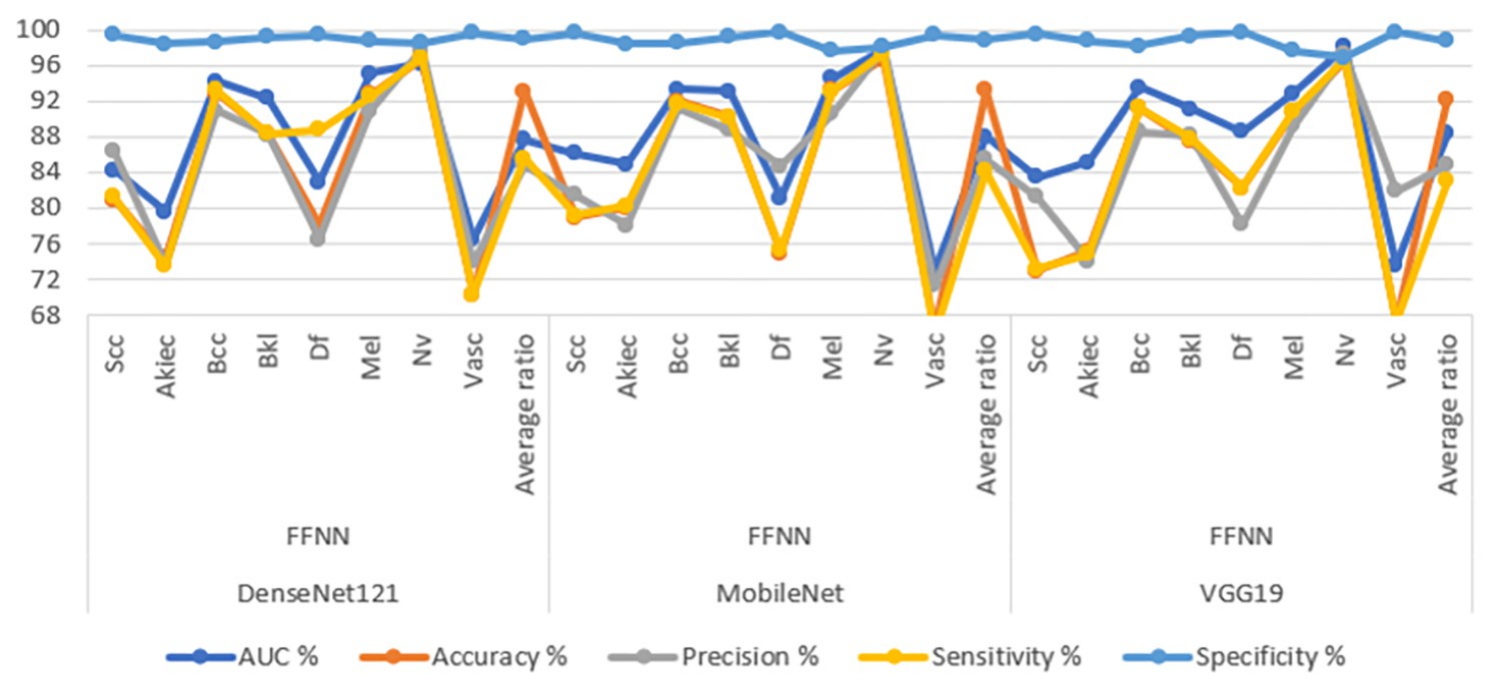
Display performance of hybrid system of CNN-FFNN for image analysis of the ISIC2019 dataset for early detection of skin lesions.

Table 5 and Fig 9 comprehensively present outcomes using employing CNN-FFNN hybrid models to analyse images sourced from the ISIC 2019 dataset for the early detection of skin lesions. The ensuing details elaborate on the results obtained for each model within this hybrid framework. The CNN-FFNN hybrid model, upon evaluation, has demonstrated exceptional performance. The DenseNet121-FFNN model achieved an AUC of 87.68%, accuracy of 93.1%, precision of 84.81%, sensitivity of 85.63%, and specificity of 99.06%. The MobileNet-FFNN model achieved an AUC of 88%, accuracy of 93.3%, precision of 85.53%, sensitivity of 84.15%, and specificity of 98.89%. The VGG19-FFNN model yielded an AUC of 88.33%, accuracy of 92.2%, precision of 84.84%, sensitivity of 83.01%, and specificity of 98.76%.

**Table 5:** Results of the hybrid system of CNN-FFNN for diagnosis of skin cancer.

Combined features	Classifier	Classes	AUC %	Accuracy %	Precision %	Sensitivity %	Specificity %
DenseNet121	FFNN	Scc	84.3	80.9	86.4	81.3	99.5
		Akiec	79.6	74.4	73.7	73.5	98.5
		Bcc	94.3	92.9	90.9	93.2	98.7
		Bkl	92.4	88.4	88.3	88.4	99.2
		Df	82.9	78.1	76.5	88.8	99.5
		Mel	95.1	92.8	90.9	92.6	98.8
		Nv	96.3	96.8	97.8	96.9	98.6
		Vasc	76.5	70.3	74	70.3	99.7
		**Average ratio**	**87.68**	**93.1**	**84.81**	**85.63**	**99.06**
MobileNet	FFNN	Scc	86.1	78.9	81.5	79.2	99.7
		Akiec	84.9	80.1	78.1	80.3	98.5
		Bcc	93.4	92	91.3	91.8	98.6
		Bkl	93.1	90.3	88.8	90.2	99.2
		Df	81.2	75	84.7	75.3	99.8
		Mel	94.6	93.3	90.6	93.1	97.7
		Nv	97.8	96.7	97.9	97.4	98.1
		Vasc	72.9	66.3	71.3	65.9	99.5
		**Average ratio**	**88**	**93.3**	**85.53**	**84.15**	**98.89**
VGG19	FFNN	Scc	83.5	72.9	81.3	73.2	99.6
		Akiec	85.2	75.2	74.1	74.8	98.8
		Bcc	93.6	91.2	88.6	91.2	98.2
		Bkl	91.1	87.5	88.1	87.8	99.3
		Df	88.6	82.3	78.2	82.3	99.8
		Mel	92.9	90.8	89.3	90.9	97.7
		Nv	98.1	96.6	97.2	96.7	96.9
		Vasc	73.6	67.3	81.9	67.2	99.8
		**Average ratio**	**88.33**	**92.2**	**84.84**	**83.01**	**98.76**

**Fig 10:**
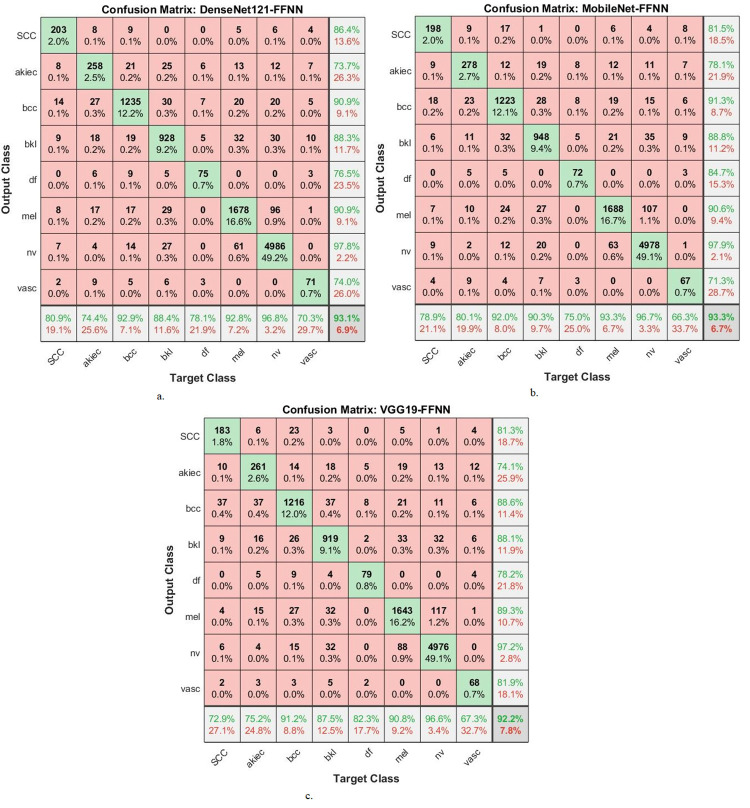
Confusion matrix for Display performance of hybrid system of CNN-FFNN for dermoscopy image analysis of the ISIC2019 dataset for early detection of skin lesions a. DenseNet121- FFNN b. MobileNet- FFNN c. VGG19- FFNN.

Fig 10 provides a graphical representation of the confusion matrices generated by the DenseNet121-FFNN, MobileNet-FFNN, and VGG19-FFNN models for early diagnosis of skin lesions. The figure furnishes insight into the accuracy attained by each class within the classification framework. Specifically, the DenseNet121-FFNN model achieves a remarkable accuracy across all classes, with the following results: 80.9% accuracy for the Scc class, 74.4% for Akice class, 92.9% for Bcc class, 88.4% for Bkl class, 78.1% for Df class, 92.8% for Mel class, 96.8% for Nv class, and 70.3% for Vasc class. Similarly, the MobileNet-FFNN model displays an accuracy across all classes. It achieves 78.9% accuracy for the Scc class, 80.1% for Akice class, 92% for Bcc class, 90.3% for Bkl class, 75% for Df class, 93.3% for Mel class, 96.7% for Nv class, and 66.3% for Vasc class. Furthermore, the VGG19-FFNN model portrays perfect accuracy rates for each class. This is evident from the results, where it obtains 72.9% accuracy for the Scc class, 75.2% for Akice class, 91.2% for Bcc class, 87.5% for Bkl class, 82.3% for Df class, 90.8% for Mel class, 96.6% for Nv class, and 67.3% for Vasc class.

### 4.6. Results of hybrid systems based on combined CNN features

The segment details the outcomes of employing hybrid systems that use combined features from DenseNet121, MobileNet, and VGG19 models. These systems are designed for the early identification of skin cancers and discrimination of such types from other types of skin lesions. The operational process of this technique involves: enhancing the image quality and then delineating the region containing the lesion. The images of lesion areas are inputted into CNN models. These images undergo a process of feature extraction through convolutional layers and pooling, thereby generating feature maps. In order to capture pertinent features while eliminating extraneous ones, these features are then transformed into a lower-dimensional space using t-SNE. The ensuing feature vectors, which result from the t-SNE transformation, are subsequently utilized for training and evaluating the performance of RF and FFNN models. By utilizing these models, the hybrid system becomes adept at effectively distinguishing between different skin conditions and cancers.

**Fig 11:**
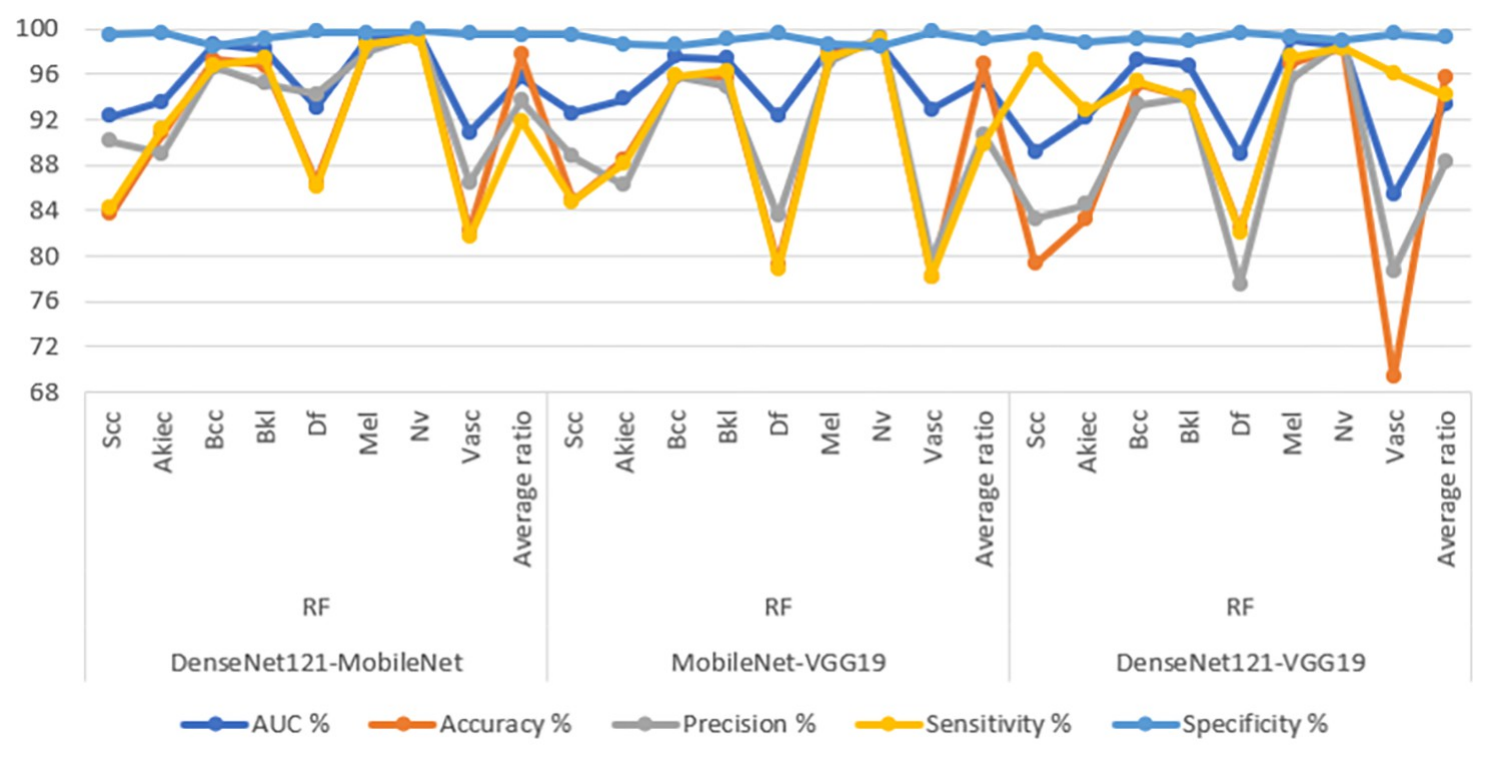
Display of results of the CNN-RF hybrid system with combined CNN model features for early diagnosis of skin lesions.

Table 6 and Fig 11 display the evaluation outcomes of the hybrid CNN-RF models, which are constructed by amalgamating features from different CNN models and classified using RF method. These models are applied to analyze images from the ISIC 2019 dataset, with the primary objective of achieving early identification of skin lesions. The ensuing results detail the performance metrics of the CNN-RF models when utilizing the combined CNN features. The model that combines features of DenseNet121 and MobileNet, referred to as DenseNet121-MobileNet-RF, showcases notable performance metrics. It attains an AUC of 95.7%, denoting the effectiveness of its discriminative capability. Moreover, its accuracy reaches 97.7%, reflecting the proportion of correctly classified instances. The precision score, which signifies the ratio of true positive predictions to the entirety of positive predictions of 93.65%. Sensitivity, also known as true positive rate, signifies the model’s ability to correctly identify positive cases and achieves a value of 91.93%. Lastly, specificity, representing the true negative rate, indicates the model’s proficiency in correctly recognizing negative cases, attaining a value of 99.49%. The next hybrid model, named MobileNet-VGG19-RF, which combined features of MobileNet with VGG19 and classifyed using RF. It achieves an AUC of 95.45%, indicating its strong discriminatory performance. The model’s accuracy reaches 96.9%, while its precision, sensitivity, and specificity values are 90.68%, 89.86%, and 99.06% respectively. The third hybrid model, named DenseNet121-VGG19-RF, combined features of DenseNet121 and VGG19. Its AUC of 93.44%, highlighting its ability to discriminate between classes. The accuracy of this model is 95.8%, while precision, sensitivity, and specificity values are 88.23%, 94.19%, and 99.26% respectively. These outcomes collectively underscore the effectiveness of the hybrid CNN-RF models in the realm of early diagnosis of skin lesions, as well as their proficiency in distinguishing between different skin conditions. The presented metrics provide a comprehensive assessment of the models’ performance and their potential clinical utility.

**Table 6:** Results of the CNN-RF hybrid system with combined CNN model features for early diagnosis of skin lesions.

Combined features	Classifier	Classes	AUC %	Accuracy %	Precision %	Sensitivity %	Specificity %
DenseNet121-MobileNet	RF	Scc	92.3	83.7	90.1	84.3	99.5
		Akiec	93.6	90.8	89	91.2	99.7
		Bcc	98.7	97.3	96.6	96.8	98.5
		Bkl	98.2	96.8	95.2	97.4	99.2
		Df	93.1	86.5	94.3	86.1	99.8
		Mel	99.2	98.5	98	98.7	99.7
		Nv	99.6	99.3	99.5	99.2	99.9
		Vasc	90.9	82.2	86.5	81.7	99.6
		**Average ratio**	**95.7**	**97.7**	**93.65**	**91.93**	**99.49**
MobileNet-VGG19	RF	Scc	92.6	84.9	88.8	84.8	99.5
		Akiec	93.8	88.5	86.2	88.2	98.7
		Bcc	97.6	95.8	95.8	95.9	98.6
		Bkl	97.4	95.8	94.9	96.3	99.1
		Df	92.3	79.2	83.5	78.8	99.6
		Mel	98.4	97.7	97.1	97.6	98.7
		Nv	98.6	99	99.3	99.1	98.5
		Vasc	92.9	78.2	79.8	78.2	99.8
		**Average ratio**	**95.45**	**96.9**	**90.68**	**89.86**	**99.06**
DenseNet121-VGG19	RF	Scc	89.2	79.3	83.3	97.3	99.6
		Akiec	92.2	83.3	84.5	92.8	98.8
		Bcc	97.3	95.1	93.3	95.4	99.2
		Bkl	96.8	94	94	93.9	98.9
		Df	88.9	82.3	77.5	82.1	99.7
		Mel	99.1	97	95.7	97.6	99.3
		Nv	98.6	98.3	98.8	98.3	99
		Vasc	85.4	69.3	78.7	96.1	99.6
		**Average ratio**	**93.44**	**95.8**	**88.23**	**94.19**	**99.26**

**Fig 12:**
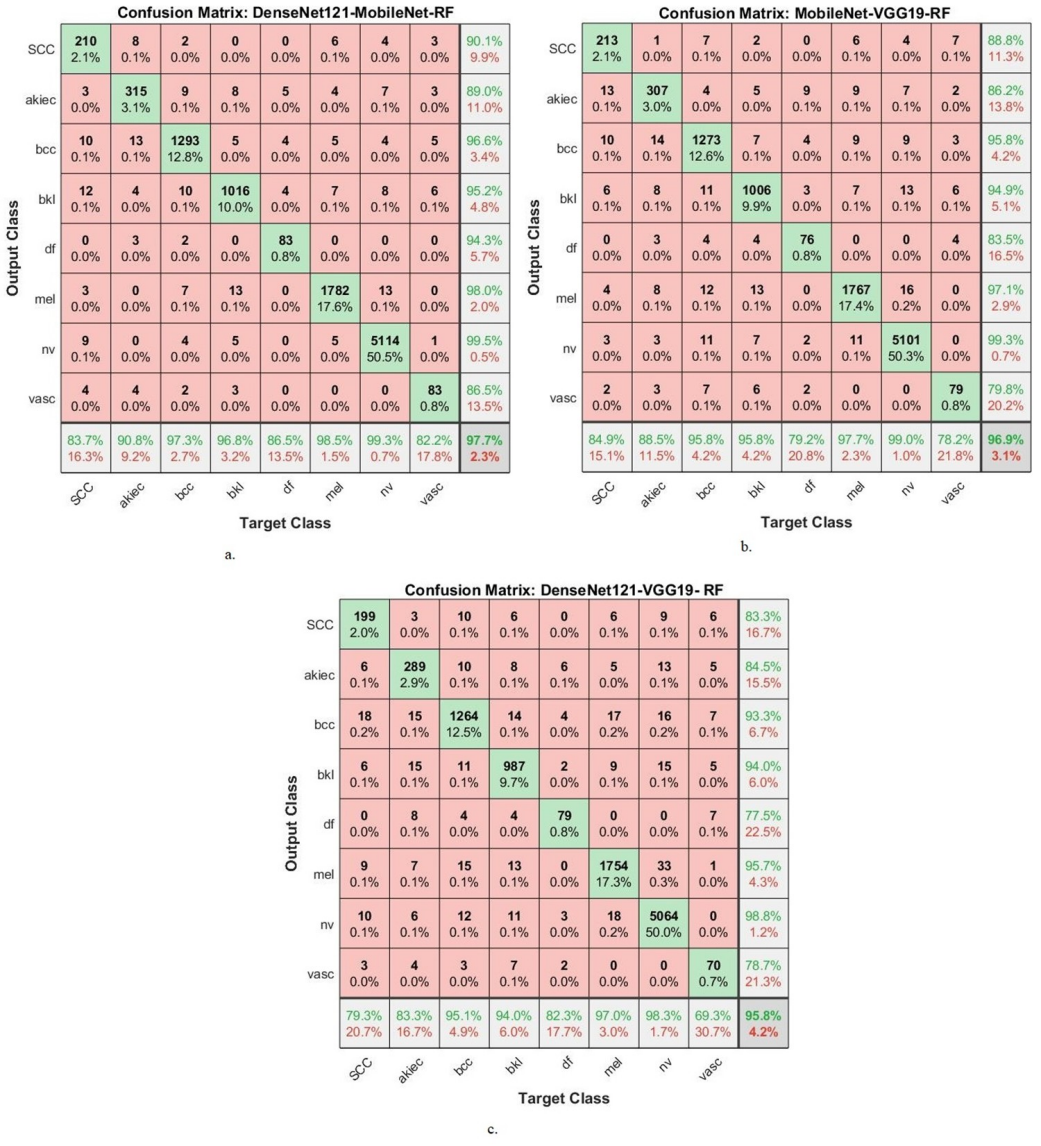
Confusion matrix for Display performance of RF network with fusion CNN features for dermoscopy image analysis of the ISIC2019 dataset for early detection of skin lesions a. DenseNet121-MobileNet-RF b. MobileNet-VGG19-RF c. DenseNet121-VGG19-RF.

Fig 12 presents a depiction of the confusion matrices generated by the hybrid models DenseNet121-MobileNet-RF, MobileNet-VGG19-RF, and DenseNet121-VGG19-RF for the early diagnosis of skin lesions. The figure offers a graphical insight into the accuracy achieved by each individual class within the classification framework. The performance of these models across various classes is detailed below. The DenseNet121-MobileNet-RF model achieves a notable level of accuracy across all classes, with the following specific accuracies: 83.7% for the Scc class, 90.8% for the Akice class, 97.3% for the Bcc class, 96.8% for the Bkl class, 86.5% for the Df class, 98.5% for the Mel class, 99.3% for the Nv class, and 82.2% for the Vasc class. Similarly, the MobileNet-VGG19-RF model also demonstrates accuracy across all classes. Specifically, its accuracy rates are as follows: 84.9% for the Scc class, 88.5% for the Akice class, 95.8% for the Bcc class, 95.8% for the Bkl class, 79.2% for the Df class, 97.7% for the Mel class, 99% for the Nv class, and 78.2% for the Vasc class. Furthermore, the DenseNet121-VGG19-RF model showcases impeccable accuracy rates for each individual class. This is evident from the following results: 79.3% accuracy for the Scc class, 83.3% for the Akice class, 95.1% for the Bcc class, 94% for the Bkl class, 82.3% for the Df class, 97% for the Mel class, 98.3% for the Nv class, and 69.3% for the Vasc class. These outcomes collectively provide an in-depth understanding of the accuracy levels achieved by each class within the classification scheme, thereby highlighting the models’ proficiency in correctly categorizing different skin lesions. The graphical representation of the confusion matrices enhances the interpretability of these results and offers valuable insights into the diagnostic capabilities of the hybrid models.

**Fig 13:**
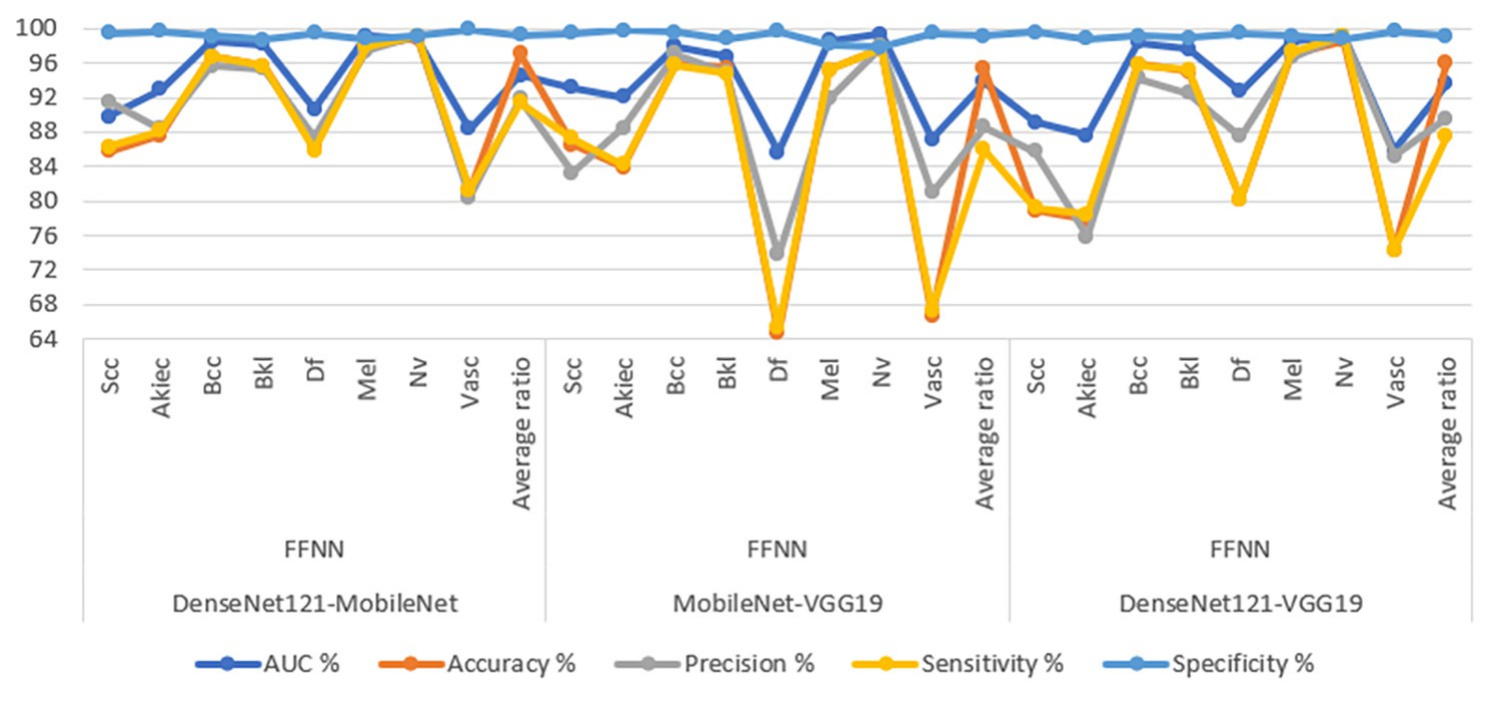
Display of results of the CNN-FFNN hybrid system with combined CNN model features for early diagnosis of skin lesions.

Table 7 and Fig 13 illustrate the evaluation results obtained from the hybrid CNN-FFNN models. These models are created by merging features from different CNN architectures and are subsequently classified using the FFNN method. The application of these models involves the analysis of images from the ISIC 2019 dataset, with a primary aim of achieving early detection of skin lesions. The ensuing findings provide a detailed overview of the performance metrics of the CNN-FFNN models when combined CNN features. One of these models, denoted as DenseNet121-MobileNet-FFNN amalgamates features from DenseNet121 and MobileNet. This model exhibits noteworthy performance indicators. Notably, it achieves an AUC of 94.55%, which signifies its effective discriminatory ability. Furthermore, the accuracy of the model reaches 97.1%, representing the proportion of correctly classified instances. Precision, indicating the ratio of true positive predictions to all positive predictions, of 91.9%. Sensitivity, or the true positive rate, attains a value of 91.39%, denoting the model’s capacity to accurately identify positive cases. Additionally, the model displays a high specificity of 99.3%, reflecting its proficiency in correctly recognizing negative cases. The subsequent hybrid model, termed MobileNet-VGG19-FFNN, combines features from MobileNet and VGG19 and is classified using the FFNN method. It achieves an AUC of 93.84%, underscoring its robust discriminatory performance. The model’s accuracy reaches 95.3%, while precision, sensitivity, and specificity values stand at 88.54%, 85.95%, and 99.11% respectively. Furthermore, the DenseNet121-VGG19-FFNN model, which combines features from DenseNet121 and VGG19, demonstrates an AUC of 93.61%, emphasizing its capacity to distinguish between different classes. The model’s accuracy is 96%, and its precision, sensitivity, and specificity values are 89.59%, 87.44%, and 99.2% respectively. Collectively, these outcomes highlight the effectiveness of the hybrid CNN-FFNN models in achieving early skin lesion diagnosis and their proficiency in accurately differentiating between various skin conditions.

**Table 7:** Results of the CNN-FFNN hybrid system with combined CNN model features for early diagnosis of skin lesions.

Combined features	Classifier	Classes	AUC %	Accuracy %	Precision %	Sensitivity %	Specificity %
DenseNet121-MobileNet	FFNN	Scc	89.8	85.7	91.5	86.3	99.5
		Akiec	92.9	87.6	88.4	88.1	99.7
		Bcc	98.5	96.8	95.7	96.8	99.1
		Bkl	98.2	95.7	95.3	95.7	98.7
		Df	90.6	86.5	87.4	85.9	99.5
		Mel	99.2	97.9	97.4	97.9	98.8
		Nv	98.8	98.9	99.1	99.1	99.2
		Vasc	88.4	81.2	80.4	81.3	99.9
		**Average ratio**	**94.55**	**97.1**	**91.9**	**91.39**	**99.3**
MobileNet-VGG19	FFNN	Scc	93.2	86.5	83.2	87.3	99.5
		Akiec	92.1	83.9	88.5	84.2	99.8
		Bcc	97.9	95.9	97.1	95.8	99.6
		Bkl	96.7	95.4	94.9	94.8	98.8
		Df	85.6	64.6	73.8	65.3	99.7
		Mel	98.6	95.2	91.9	95.2	98.2
		Nv	99.4	97.6	97.9	97.7	97.8
		Vasc	87.2	66.7	81	67.3	99.5
		**Average ratio**	**93.84**	**95.3**	**88.54**	**85.95**	**99.11**
DenseNet121-VGG19	FFNN	Scc	89.1	78.9	85.7	79.2	99.6
		Akiec	87.6	77.8	75.8	78.4	98.8
		Bcc	98.4	95.9	94.2	95.8	99.2
		Bkl	97.6	95	92.5	95.2	98.9
		Df	92.7	80.2	87.5	80.2	99.5
		Mel	98.5	97	96.6	97.3	99.1
		Nv	99.1	98.6	99.2	99.1	98.8
		Vasc	85.9	74.3	85.2	74.3	99.7
		**Average ratio**	**93.61**	**96**	**89.59**	**87.44**	**99.2**

**Fig 14:**
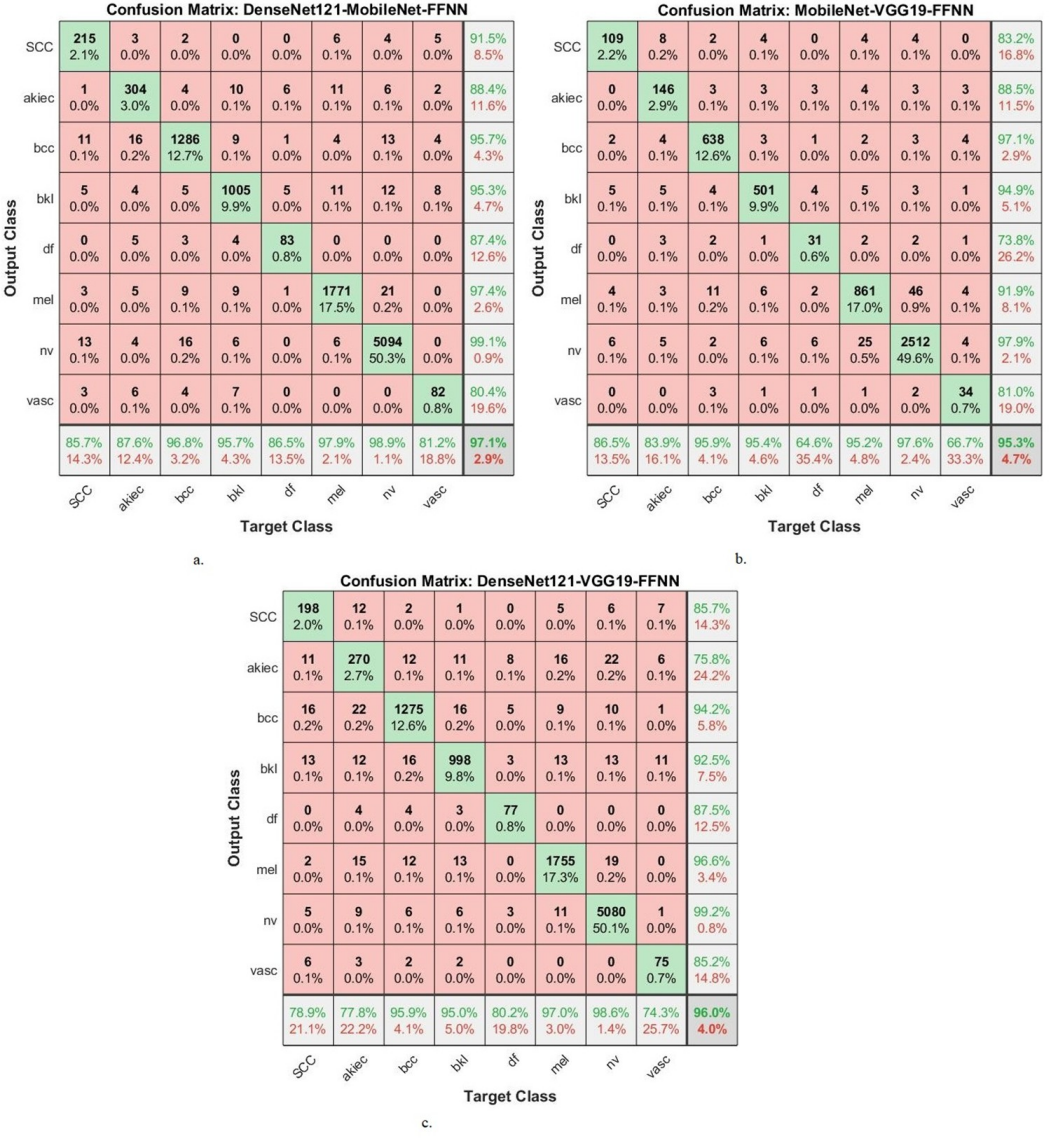
Confusion matrix for Display performance of FFNN network with fusion CNN features for dermoscopy image analysis of the ISIC2019 dataset for early detection of skin lesions a. DenseNet121-MobileNet-FFNN b. MobileNet-VGG19-FFNN c. DenseNet121-VGG19-FFNN.

Fig 14 illustrates a graphical representation of the confusion matrices generated by the hybrid models DenseNet121-MobileNet-FFNN, MobileNet-VGG19-FFNN, and DenseNet121-VGG19-FFNN for the purpose of early skin lesion diagnosis. The figure provides a visual insight into the accuracy attained by each specific class within the classification framework. The performance of these models across the various classes is elaborated below. The DenseNet121-MobileNet-FFNN model achieves a significant degree of accuracy across all classes. The specific accuracy rates are as follows: 85.7% for the Scc class, 87.6% for the Akice class, 96.8% for the Bcc class, 95.7% for the Bkl class, 86.5% for the Df class, 97.9% for the Mel class, 98.9% for the Nv class, and 81.2% for the Vasc class. Similarly, the MobileNet-VGG19-FFNN model also demonstrates accuracy across all classes. Its specific accuracy rates are as follows: 86.5% for the Scc class, 83.9% for the Akice class, 95.9% for the Bcc class, 95.4% for the Bkl class, 64.6% for the Df class, 95.2% for the Mel class, 97.6% for the Nv class, and 66.7% for the Vasc class. Furthermore, the DenseNet121-VGG19-FFNN model exhibits remarkable accuracy rates for each individual class. This is evident from the following outcomes: 78.9% accuracy for the Scc class, 77.8% for the Akice class, 95.9% for the Bcc class, 95% for the Bkl class, 80.2% for the Df class, 97% for the Mel class, 98.6% for the Nv class, and 74.3% for the Vasc class.

These findings collectively provide a comprehensive understanding of the achieved accuracy levels for each class within the classification scheme. This emphasizes the adeptness of the models in accurately categorizing diverse skin lesions. The graphical depiction of the confusion matrices enhances the interpretability of these results and offers valuable insights into the diagnostic proficiency of the hybrid models. In this study, employed two strategies for the analysis and diagnosis of skin lesions of dermoscopy images from the ISIC2019 dataset. These strategies involve the integration of features from various CNN models (DenseNet121, MobileNet, and VGG19) along with the utilization of RF and FFNN algorithms.

## 5. Discussion and comparison performance systema

Skin lesions are abnormalities or changes in the skin’s appearance, texture, or color that indicate skin cancer or other skin diseases. Dermoscopy is a diagnostic technique that involves examining these skin lesions under a magnifying instrument to aid in their accurate diagnosis. The ISIC2019 dataset comprises a collection of dermoscopy images that serve as a valuable resource for studying and diagnosing skin lesions. Early and accurate diagnosis is crucial for effective treatment, as it prevent potential complications and ensure better patient outcomes. AI techniques have emerged as powerful tools in the field of dermatology for the early diagnosis of various skin lesions. AI methods, such as CNNs, RF, and FFNN are being employed to analyze dermoscopy images and aid in the identification of similar types of skin lesions. By training these AI models on large datasets like ISIC2019, they learn to recognize patterns and features that are easily discernible to the human eye. This enables them to assist dermatologists in making accurate and timely diagnoses, potentially leading to faster and more effective treatments.

In the first strategy, introduced a hybrid approach that combines the strengths of CNN models with RF and FFNN algorithms to enhance the accuracy of skin lesion diagnosis. Specifically, evaluated the performance of DenseNet121-RF, MobileNet-RF, and VGG19-RF models, which achieved accuracies of 93.6%, 92.8%, and 92.6% respectively. When inputting the features extracted from DenseNet121, MobileNet, and VGG19 into the FFNN network, observed accuracies of 93.1%, 93.3%, and 92.2% respectively. Moving on to the second strategy, our focus shifted towards an integrated approach based on the fusion of CNN model features for dermoscopy image analysis and skin lesion diagnosis. By adopting this strategy, harnessed the combined potential of CNN models to achieve accurate results. The RF network, leveraging the hybrid features of DenseNet121-MobileNet, MobileNet-DenseNet121, and DenseNet121-VGG19 models, achieved impressive accuracy levels of 97.7%, 96.9%, and 95.8% respectively. On the other hand, the FFNN approach, utilizing hybrid features from the same models, demonstrated accuracy rates of 97.1%, 95.3%, and 96.0%. Comparing the two strategies, observe that the hybrid strategy focusing on the integration of features from CNN models for dermoscopy image analysis yielded higher accuracy levels in the diagnosis of skin lesions. The RF and FFNN networks displayed strong performance in both strategies, with accuracy levels consistently surpassing the 90% mark. Notably, the second strategy, which emphasized the fusion of CNN model features, produced higher accuracy rates in most cases. This highlights the potential of feature integration and the robustness of these hybrid models in accurately identifying and diagnosing different types of skin lesions. This study underscores the effectiveness of leveraging hybrid approaches that integrate features from CNN models, RF, and FFNN algorithms for the early diagnosis of skin lesions in dermoscopy images. The integration of CNN model features appears to offer a promising avenue for improving accuracy, and further exploration of these hybrid strategies could lead to enhanced diagnostic capabilities in clinical practice.

Compared to previous studies, our strategies focused on leveraging the power of hybrid models combining CNN architectures with RF and FFNN algorithms to enhance the accuracy of early skin lesion diagnosis from dermoscopy images. Let’s compare our results with those of the previously mentioned studies as shown in Table 8. Based on the results summarized in the table, the proposed hybrid system integrating DenseNet121-MobileNet-RF achieves excellent performance exceeding most prior methods for diagnosing skin lesions. The proposed system attains an AUC of 95.7%, accuracy of 97.7%, precision of 93.65%, sensitivity of 91.93% and specificity of 99.49%. This outperforms earlier approaches including InSiNet, ROI extraction, DCNN, Swin Transformer +CNN, weighted model ensembles, and gradient visualization methods in terms of overall accuracy and other metrics. The use of optimized feature fusion from DenseNet121 and MobileNet, followed by the RF classifier enables the proposed method to accurately distinguish different skin conditions. By integrating the strengths of CNN models with RF and FFNN algorithms, achieved higher accuracy levels, highlighting the potential of our approach for enhancing early skin lesion diagnosis and clinical decision-making.

**Table 8:** Comparison of the performance of previous systems with the proposed systems for diagnosing skin lesions.

Author	Technique/Model	Results
Hatice et al. [13]	InSiNet (CNN)	Accuracy: 91.89%
Seyed et al. [14]	Ensemble (CNNs + Texture Features)	Accuracy: 96.7%, Avg Precision: 95.1%, Sensitivity: 96.3%, Specificity: 97.1%
Hadi et al. [15]	Preprocessing for RoI extraction	Accuracy Improvement: 2.18% (Using InceptionResNetV2)
Syed et al. [17]	CNN with surrogate gradient descent	Accuracy: 89.57% (Outperformed VGG-13 and AlexNet)
Imran et al. [19]	DCNN	Precision: 94%, Specificity: 91%, Sensitivity: 93%
Selen et al. [20]	Swin Transformer + CNNs	Sensitivity: 82.3%, Specificity: 97.9%, Accuracy: 97.2%
Zillur et al. [21]	Weighted Average Model	Macro-average recall: 91%, 84% (DenseNet, Xception, ResNet)
Irfan et al. [23]	Ensemble (EfficientNetB3 + XGBoost)	Accuracy: 78%, Precision: 89%, Recall: 86%, F1 Score: 88%
Qilin et al. [24]	Grad-CAM for Dermoscopic Images	Balanced Multiclass Accuracy: 88.7%
Natasha et al. [25]	Explainable AI System	Accuracy: 94.47%, Recall: 94.01%
Arias et al. [26]	CNNs with Transfer Learning	Accuracy: 93%
Proposed System	hybrid system of DenseNet121-MobileNet-RF	AUC of 95.7%, an accuracy of 97.7%, a precision of 93.65%, a sensitivity of 91.93%, and a specificity of 99.49%

## 6. Conclusions

Skin lesions often share similarities in their early stages, posing challenges in distinguishing between skin cancers and other types of lesions. As a result, numerous hybrid systems have emerged, leveraging combined features to address this issue. These systems improve the images and subsequently segment the affected area from the healthy skin using the GVF algorithm. The extracted lesions regions are then inputted into DenseNet121, MobileNet, and VGG19 models. These models are pivotal in extracting features and developing two hybrid systems designed for early skin cancer diagnosis and differentiation from other skin lesions. The first hybrid approach involves the integration of CNN models with RF and FFNN algorithms. The second hybrid strategy focused on the fusion of CNN features (DenseNet121-MobileNet, MobileNet-VGG19 and DenseNet121-VGG19) and inputted into RF and FFFNN networks. Remarkably, the hybrid system of DenseNet121-MobileNet-RF achieved remarkable performance metrics, including an AUC of 95.7%, an accuracy of 97.7%, a precision of 93.65%, a sensitivity of 91.93%, and a specificity of 99.49%. This comprehensive approach demonstrates the potential of hybrid systems, feature fusion, and advanced algorithms in enhancing the accuracy and effectiveness of early diagnosis and distinction of skin cancers from other skin lesions.
